# Metabolic modulation of CtBP dimeric status impacts the repression of DNA damage repair genes and the platinum sensitivity of ovarian cancer

**DOI:** 10.7150/ijbs.80952

**Published:** 2023-04-09

**Authors:** Jingjing Li, Yuan Wang, Li Wang, Dapeng Hao, Peipei Li, Minxia Su, Zhiqiang Zhao, Tianyu Liu, Lixin Tai, jinjian Lu, Li-jun Di

**Affiliations:** 1 Department of Biological Sciences, Faculty of Health Sciences, University of Macau, Macau, PR China.; 2 Cancer Center, Faculty of Health Sciences, University of Macau, Macau, PR China.; 3 Institute of Translational Medicine, Faculty of Health Sciences, University of Macau, Macau, PR China.; 4 Ministry of Education Frontiers Science Center for Precision Oncology, University of Macau, PR China.; 5 Institute of Chinese Medical Sciences, University of Macau, Macau, PR China.; 6 Current address: Jinming Yu Academician Workstation of Oncology, Affiliated Hospital of Weifang Medical University, Weifang, Shandong, PR China.; 7 Current address: State Key Laboratory of Respiratory Disease & National Clinical Research Center for Respiratory Disease, Guangzhou Institute of Respiratory Health, the First Affiliated Hospital of Guangzhou Medical University, Guangzhou Medical University, Guangzhou, PR China.

**Keywords:** Ovarian cancer, Cisplatin, CtBP, Drug resistance, Metformin

## Abstract

Platinum drug-based chemotherapy plays a dominant role in OC (ovarian cancer) treatment. The expression of DNA damage repair (DDR) genes is critical in distinguishing drug-sensitive and drug-refractory patients, as well as in the development of drug resistance in long-term treated patients. CtBP is a highly expressed oncogene in OC and was found to repress DDR genes expression in our previous study. In the present study, the formation of CtBP dimers in live cells was studied, and the functional differences between monomeric and oligomeric CtBP were explored by CHIP-seq and RNA-seq. Besides, the dynamics of CtBP dimer formation in response to the metabolic modulation were investigated by the protein fragment complementation (PCA) assays. We show that dimerized CtBP, but not the dimerization-defective mutant, binds to and represses DDR gene expression in OC cells. Treatment of the mice tumors grown from engrafted OC cells by cisplatin disclosed that high-level CtBP expression promotes the CtBP dimerization and increases the therapeutic effect of cisplatin. Moreover, the CtBP dimerization is responsive to the intracellular metabolic status as represented by the free NADH abundance. Metformin was found to increase the dimerization of CtBP and potentiate the therapeutic effect of cisplatin in a CtBP dimerization-dependent manner. Our data suggest that the CtBP dimerization status is a potential biomarker to predict platinum drug sensitivity in patients with ovarian cancer and a target of metformin to improve the therapeutic effect of platinum drugs in OC treatment.

## Background

Ovarian cancer is recognized as the leading cause of death among women with gynecological cancers, with an annual incidence of 239,000 new cases and 152,000 deaths worldwide 1, 2. Epithelial ovarian cancer (EOC) is the most common pathological type, with serous ovarian cancer (SOC) identified as the main subtype that is originated from either fallopian tube or ovary 3. EOC usually has a very poor prognosis and the estimated five-year survival rate of patients with EOC is less than 50%, with a relapse rate of 70% to 80% 4.

Chemotherapy is still the first choice for advanced EOC treatment in addition to surgical debulking of the primary tumor. For unresectable EOC, neoadjuvant chemotherapy is recommended before interval debulking surgery. Platinum-based anticancer drugs such as cisplatin (cis-diamminedichloroplatinum, CDDP or CP), carboplatin and oxaliplatin are frequently prescribed for EOC chemotherapy. Through passive diffusion or binding to cell membrane proteins, these platinum drugs are absorbed into the cells, where these small chemicals react with the nucleotides of DNA (mainly guanine) and form platinum-DNA adducts, subsequently causing intrastrand or interstrand crosslinks. The interstrand crosslinking of DNA by platinum drugs represents the main antitumor effect because it interferes with gene expression, DNA replication, and cell mitosis 5. Consequently, these cells undergo apoptosis following the failure to repair the damage caused by these drugs.

Although the majority of patients with OC may benefit from platinum drug treatment, some patients are considered drug refractory due to their negative response to platinum drugs. For the drug-sensitive group, chemoresistance also eventually develops and becomes a major challenge in platinum-based OC therapy. The explanations for drug refractoriness and drug resistance are complicated. Intrinsically, the heterogeneity of cancer cells enables some cancer cells to escape from drugs due to inheritable mutations, which endow these cells with some benefits, such as reducing drug absorption, increasing drug detoxification, inducing apoptosis defects, and enhancing DNA repair. All the known DNA repair pathways are involved in the negative response or resistance to platinum-based chemotherapy in diverse types of cancer. Defects in any one of these DNA repair pathways will lead to compensation from the other pathways. The NER (nucleotide excision repair) DDR pathway, as represented by ERCC1 gene expression, specifically removes the platinum-DNA adduct and the intrastrand DNA crosslink by removing a bulk piece of the DNA strand. On the other hand, the HR (homologous-recombination mediated repair) pathway mainly repairs the DNA double-strand breaks (DSBs) caused by the interstrand crosslinks with platinum 6, 7. Half of patients with high-grade SOC have defects in the genes involved in HR pathways, such as ATM, ATR, BRCA1, BRCA2, RAD51C, MRN complex, Fanconi anemia (FA) genes, and CHK1/2 8, 9.

C-terminal binding protein (CtBP) is a transcriptional corepressor involved in multiple bioprocesses. Recent studies have documented that CtBP functions as an oncogene to promote cancer initiation 10-12 and metastasis 13, 14, regulate intracellular metabolism 15-17, and increase drug resistance by repressing DDR genes 18. In a pan-cancer analysis, CtBP showed the highest expression in OC. Our recent results verified that CtBP also transcriptionally inhibits DDR genes in OC by forming a complex with estrogen receptor alpha (ERα) 19.

Human possesses two CtBP gene loci, CTBP1 and CTBP2. CtBP1 and CtBP2 proteins share 78% amino acid identity and 83% similarity 20. Both CtBPs contain a conserved N-terminal domain that binds to the other proteins possessing a consensus Pro‐X‐Asp‐Leu‐Ser (PXDLS) motif and a conserved central domain with high homology to NAD^+^/NADH-dependent dehydrogenases 21, 22. The role of the catalytic domain in regulating CtBP-mediated gene transcription is controversial 21, 23, but mutations in the catalytic site compromised corepressor function in some studies 24-27. Two studies performed approximately two decades ago established that CtBP forms a dimer with the involvement of dinucleotides NAD^+^ or NADH 22, 28. Recent structural studies also showed that the CtBP dimers further oligomerize to form tetramers 29, 30. Some critical residues were found to be essential for the oligomerization of CtBP 31. In cell, CtBP is presumed to exist in an equilibrium between monomeric and oligomeric states 32 33. Some studies further proposed that oligomeric CtBP is essential for gene repression 34. Due to the higher affinity of NADH in mediating CtBP dimerization than NAD^+^, the NADH/NAD^+^ ratio has been shown to influence gene repression by CtBP in breast cancer cells 35, fibroblasts, stem cells 36 31, and other cell types. However, direct evidence showing the existence of CtBP dimers or tetramers in live cells remains elusive. Researchers have not yet determined whether CtBP dimer formation is dynamically responsive to the intracellular NADH levels, NAD^+^ levels or the NADH/NAD^+^ ratio.

In the present study, the formation of CtBP dimers in live cells was studied, and the dynamics of CtBP dimer formation in response to the metabolic status were also investigated. Further profiling of CtBP binding in OC cells identified DDR genes as target genes regulated by dimerized CtBP, but not dimerization defective CtBP mutant. Since platinum reduces the intracellular NAD^+^ and NADH pool and weakens CtBP-mediated repression of DDR genes, which fundamentally causes drug resistance in cancer cells, we provide evidence validating the hypothesis that metformin reverses the decrease in the NAD^+^/NADH ratio caused by platinum and improves drug sensitivity in OC cells.

## Methods and Materials

### Cell lines, Chemicals and Antibodies

HEK293 cells, SKOV3 cells, OVCAR-3, and ES-2 cells were all maintained in regular DMEM supplemented with 10% (v/v) FBS, penicillin-streptomycin. The antibodies applied in this study include anti-HA antibody (Beyotime, AF0039), anti-Flag antibody (Sigma F1804), anti-CtBP1 antibody (BD biosciences 612042), anti-CtBP2 antibody (BD biosciences, 612044), anti-γH2AX (Sigma-Aldrich, SAB5600038) and normal mouse IgG (Santa Cruz, sc2025). The chemicals include MTOB (4-methylthio-2-oxobutanoate, Sigma-Aldrich, k6000), 2-DG (2-Deoxy-D-glucose, Sigma-Aldrich, D8375), 3-BP (3-bromopyruvate, Sigma-Aldrich, 16490), FK866 (Sigma-Aldrich, F8557), cisplatin (Sigma-Aldrich, PHR1624), metformin (Sigma-Aldrich, PHR1084), penicillin-streptomycin (ThermoFisher), Puromycin (ThermoFisher). Luciferin (Yeasen), Doxycycline (Beyotime).

### Vectors and oligoes

The pLVX-tight-puro lentivirus vector (Clontech) was used for cloning of CtBP1 and CtBP2 as inducible lentivirus expression vector. The mutations of CtBP1 and CtBP2 was created on these vectors through the primer mediated mutagenesis procedure. The inducible CtBP1 (W318G) and CtBP2 (W324G) expression vectors pLVX-tight-puro-CtBP1 and pLVX-tight-puro-CtBP2, together with the pLVX-Tet-on Advanced, were packaged as lentivirus for infection. T293 cells was used to package the lentivirus and the supernatant was harvested post transfection for 72 hours, and spun down, filtered with 0.45μM syringe filter. The target cells were infected and further selected with Puromycin (2ug/ml) for stable cell lines. Dox (500ng/ml) was added to the cell culture medium to induce inserting gene expression. The SKOV3 cells or ES-2 cells with stable overexpression of CtBP1 (W318G) or CtBP2 (W324G) were maintained in regular DMEM supplemented with 10% (v/v) FBS, penicillin-streptomycin, 500 ng/ml puromycin. For BioID assay, CtBP was cloned into the BioID vector pcDNA3.1 MCS-BirA(R118G)-HA (addgene 36047). The EGFP-PCA reporters pBiFC-VC155 (Addgene 22011) and pBiFC-VN155(I152L) (Addgene 27097) were used for cloning the CtBP1 and CtBP2 coding sequences. The vectors for constructing Gluc-PCA reporters were kindly provided by Prof. Stephen W Michnick as gift. pcDNA3.1-Peredox-mCherry (Addgene :32383 or 32384) was applied to monitor the change of NAD+/NADH ratio. All the gene knockdown experiments were through the lentivirus vector pLKO1. The shRNAs targeting CtBP (Sense 5'-CCGGAGGGAGGACCTGGAGAAGTTCCTCGAGGAACTTCTCCAGGTCCTC-3' and anti-sense 5'- AATTCAAAAAAGGGAGGACCTGGAGAAGTTCCTCGAGGAACTTCTCCAGGTCCTCCCT-3') were cloned into pLKO1 for virus packaging in 293T cells and the supernatant were used for transduction directly or further concentration.

### Co-immunoprecipitation (Co-IP)

Treated cells were extracted with ice-cold IP buffer (150 mM Tris pH 7.5, 150 mM NaCl, 1 mM EDTA, 1 mM EGTA, 1% Triton X-100, 2.5 mM Sodium pyrophosphate, 10% glycerol, 1% NP40, 1 mM DTT) supplemented by fresh Protease inhibitor. Cell lysate was sonicated for 10 cycles with 30 s on and 30 s off using sonicator (Qsonica Q700). The Supernatant after centrifugation at 12500 rpm for 10min was used as input. Protein G agarose beads was incubated with primary antibody with gentle rocking at 4 °C for overnight, then added into input. The beads were recovered and washed with IP buffer three times with 5 mins for each wash. The bound proteins by beads were eluted by 1x PAGE loading buffer and detected by standard western blotting assay.

### BioID assay

For BioID pull down assay 37, cells were seeded into two 10-cm dishes for each experimental condition. After the transfection of CtBP-BirA*, 50 μM biotin was supplemented into the culture medium 3h post-transfection, 24h later, the cells were lysed, and the proteins were extracted in the presence of protease inhibitors. The supernatant of cell lysates was gently transferred to 2 mL tubes and was diluted to 2.5 folds with pre-chilled 50 mM Tris·Cl, pH 7.4. Subsequently the lysates were aliquoted to 1.5 mL per tube. The magnetic streptavidin beads should be equilibrium in 1:1 lysis buffer and 50 mM Tris·Cl, pH 7.4. Using the magnetic separation stand to collect the magnetic beads and remove the buffer after equilibrium. The cell extracts and beads were mixed gently, and they were incubated on the rotator overnight at 4°C. The magnetic beads were washed once sequentially by Wash Buffer 1 to 3 once and twice by Wash Buffer 4 (Wash buffer 1: 2% SDS in H2O; Wash buffer 2: 0.1% deoxycholate, 1% Triton X-100, 500 mM NaCl, 1 mM EDTA, and 50 mM Hepes, pH 7.5; Wash buffer 3: 250 mM LiCl, 0.5% NP-40, 0.5% deoxycholate, 1 mM EDTA, and 10 mM Tris, pH 8.0; Wash buffer 4: 50 mM Tris, pH 7.4). The protein is eluted from the beads for standard western blotting assay.

### Western blotting

The cells were lysed on ice using RIPA Buffer (Thermo # 89901) with the presence cocktail protease inhibitor (Sigma #SRE0055). Total protein (20 μg) from each sample was separated by SDS-PAGE in SDS running buffer (TAKARA #T9101) at 150 V for 1h at room temperature, and transferred to PVDF membranes at 300mA for 3h at 4°C. Blots were then probed with primary antibody at 1:1000 overnight at 4°C. Then the membrane was washed and incubated with HRP-conjugated secondary antibody (Santa Cruz) at 1:5000 dilution. After being washed for 5 times in PBST, the membrane was incubated with ECL detection reagent (#RPN2235) and then visualized with ChemiDoc Touch Imaging system (Bio-Rad).

### Immunofluorescence assay

Cells were grown on coverslips. The cells were washed twice with PBS and fixed in 3.5% paraformaldehyde. After washing the fixed cells with PBS for 3 times, the cells are permeabilized in PBS supplemented with 10% goat serum and 0.3% Triton X-100 for 15mins. Then the cells were treated with 3% H_2_O_2_ for 10mins and washed for twice. The cells were treated by blocking buffer (10% goat serum in 1xPBS) for 1hr. The cells were incubated with primary antibody (1:50, anti-γH2AX) in blocking buffer for 1.5hr. After washing, the cells were incubated with Alexa Fluor® 488 Goat Anti-Mouse IgG for 1hr. After washing the cells for 3 times in PBS, the cover slips were mounted using VECTASHIELD with DAPI.

### PCA (protein complementation assay)

The EGFP-PCA reporters pBiFC-VC155 (Addgene 22011) and pBiFC-VN155(I152L) (addgene 27097) were used for cloning the CtBP1 and CtBP2 coding sequences. Each vector has build-in coding sequence for the N-terminal EGFP and C-terminal EGFP, and the appropriate cloning site for target proteins. Similarly, the Gluc assay was established based on the splitting of Glussia luciferase to N-Gluc and C-Gluc. The vectors for constructing Gluc-PCA reporters were kindly provided by Prof. Stephen W Michnick as gift. CtBP2 coding sequence was cloned into N-Gluc and C-Gluc vectors respectively.

### ChIP and ChIPseq

Cells were cross-linked with 1% (w/v) formaldehyde for 5 min at room temperature. Ice cold glycine (125mM) was applied to quench formaldehyde. Then the cells were washed twice with ice cold PBS and collected. Cross-linked cells were resuspended in 1 ml immunoprecipitation (IP) buffer (150mMNaCl, 50mM Tris-HCl (pH 7.5), 5mM EDTA, NP-40(0.5%), Triton X-100 (1%), and added cocktail proteinase inhibitor (Sigma). Cell lysate were sonicated for 10 x 30 s with 30 s break using Qsonica Q700 sonicator. Then the sonicated cells were centrifuged and the supernatant was performed for immunoprecipitation. Each antibody was incubated with lysate overnight with rotation at 4 °C. And then the lysate was incubated with pre-blocked protein G beads with rotation for 10h at 4 °C. Then the beads were rinsed with high salt IP buffer supplemented with 500mM NaCl, IP buffer and finally resuspended in TE buffer (pH 8.0). Then Proteinase K (Qiagen #1018832) was added in DNA-protein complex for digestion overnight at 65 °C. Finally, the DNA was purified by phenol-chloroform extraction and ethanol precipitation with the presence of glycogen (Ambion #AM9510). The purified DNA was used for real-time PCR or library construction. The library preparation followed protocol of NEBNext® Ultra™ II DNA Library Prep Kit for Illumina® (E7645). The DNA was selected by the SPRIselect beads (Beckman). The library quality was also checked by Agilent bioanalyzer 2100.

### ChIP-seq data processing

Raw reads were trimmed by 'trimmomatic' with following parameter: HEADCROP 10, CROP 100, SLIDINGWINDOW:5:25, MINLEN:25. Bowtie2 was used to mapping the trimmed reads to hg19 genome reference with default parameter. The low mapping quality reads were removed with -q 30 by samtools. And the total mapping read of different samples were scaled to the same by subsample process. The CTBP binding peaks were called with MACS2. Peak annotation was performed with R package “ChIPpeakAnno” and the promoter region was defined as upstream 1000bp and downstream 500bp of TSS. “ChromPlot” was performed to show the peak distribution across the genome for WT and MT samples. The ChIP-seq data are provided as peaks in Supplementary Table 1 and Supplementary Table 2.

### RNA-seq data processing

Data was mapping to hg19 with Tophat2 and gene expression FPKM was obtained with Cufllinks. Default parameters were used for these tools. Differential expressed genes were defined as genes with 1.5-fold change differences. CtBP2 affected genes were defined as the genes that show differential expression between CtBP2 and CtBP2 mutant, and at the same time, show differential CtBP2 or CtBP2 mutant binding signal on gene promoter region (> 1.5 fold-change). Gene expression data and clinical information of TCGA OV samples were obtained from cBioportal website. The R package 'ConsensusClusterPlus' was used to perform the consensus clustering on TCGA OV samples with all the expression of CtBP2 mutant affected genes. The functional enrichment analysis was done with 'clusterProfiler' and 'homer' was used to perform the motif analysis. The gene expression data are provided in Supplementary Table 3 and Supplementary Table 4.

### NADH/NAD+ fluorescence intensity detection

HEK293 cells were transfected with pcDNA3.1-Peredox-mCherry (addgene 32383 or 32384) plasmid, then the cells were treated by indicated drugs. Peredox and mCherry fluorescence signal were captured with Zeiss LSM710 confocal microscopy. Excitation of Peredox was by a 405 nm laser and the emission was captured via the FITC filter (520 nm) bandpass excitation filter. mCherry was excited via a 550 laser and the emission was captured by the TriTC filter (570 nm). Images were taken and Image J was used to analyze the intensity of each cell.

### Seahorse cell metabolic detection

Glycolysis stress and mitochondria respiration stress assays were performed according to standard protocol of Agilent seahorse XF cell procedure. In brief, we seeded 5 × 10^4 cells in XF24 cell culture microplate and the cells were incubated for 12 h at 37 °C and 5% CO2. Before applying microplate to Agilent seahorse XF 24 extracellular Flux Analyzer, the culture medium was replaced with glucose free medium. The glycolysis stress test data analysis was based on the standard protocol.

### Animal experiment

All the animal experiments were approved by the UMARE (Animal Research Ethics Committee of University of Macau). Female NOD-SCID mice (6-8 weeks old) were subcutaneously injected with 5 × 10^6^ ES-2 cells (labelled by luciferase expression cassette, Addgene 72485) in each hind limb and randomly divided into different experimental groups 5 weeks after cancer cells injection. Mice were administrated with solvent or drugs by intraperitoneal injection every 3 days (cisplatin 5 mg/kg, metformin 250 mg/kg). Tumors were imaged by In-Vivo Xtreme (Bruker) by peritoneal injection of luciferin. Finally, the tumors were surgically removed for further analysis.

### Statistical analysis

Statistical analysis was performed using Student's t-test except otherwise specified. p < 0.05 was considered significant.

## Results

### Live cell monitoring of CtBP dimerization using PCA assay

**Figure 1:**
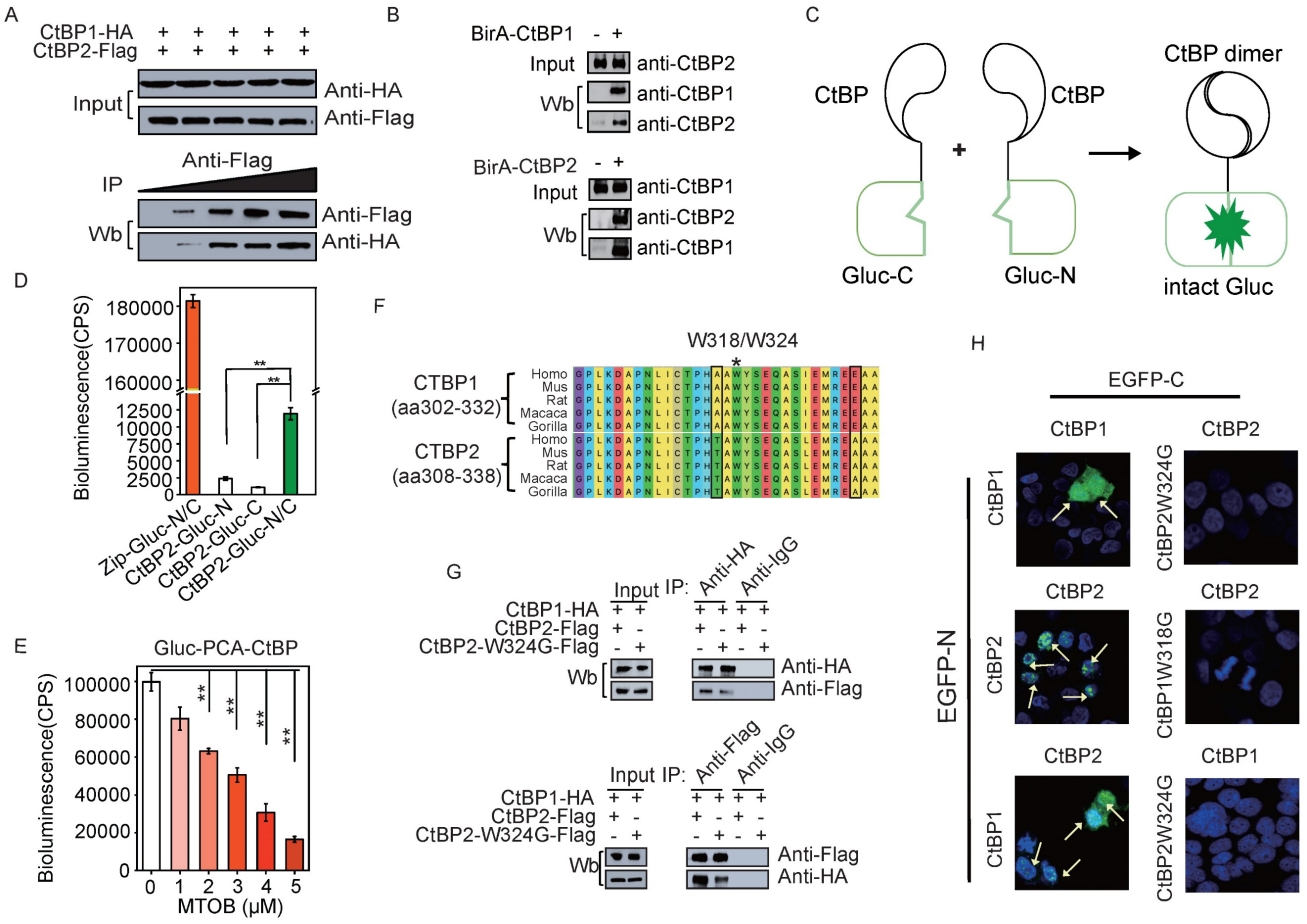
** CtBP dimerization in living cells.** A. Co-IP assay of the CtBP1-HA and CtBP2-Flag interaction in SKOV3 cells. An increasing amount of anti-Flag antibody was applied (from 0 µg/ml to 1 µg/ml). B. BioID assay of BirA-CtBP1 (top panel) and BirA-CtBP2 (bottom panel) interacting proteins. Western blotting was employed to confirm the presence of the targeted interacting proteins (both CtBP1 and CtBP2). C. A model depicting the mechanism of Gluc-PCA. D. Detection of the CtBP2 homodimer by the Gluc-PCA reporter. Zip-Gluc-N/C serves as a positive control 41. CtBP2-Gluc-C and CtBP2-Gluc-N are two fusion proteins, with each containing half of the Gluc protein. Two biological replicates (r1 and r2) are shown for co-transfection of CtBP2-Gluc-N and CtBP2-Gluc-C in 293 cells. The error bars represent the SD of three biological replicates. ** p<0.01. E. Gluc-PCA assay to analyze the response of CtBP dimerization to MTOB in 293 cells. The error bars represent the SD of three biological replicates. ** p<0.01. F. The diagram depicts the highly conserved region containing the residues in both CtBP1 and CtBP2 in which the dimerization-defective mutation was introduced. G. Pull-down assay in 293 cells co-transfected with CtBP1-HA and CtBP2-Flag/CtBP2-W324G-Flag (top panel: pull-down with an anti-HA antibody, bottom panel: pull-down with an anti-Flag antibody). H. Detection of the CtBP1 homodimer, CtBP2 homodimer, and CtBP1/CtBP2 heterodimer using the EGFP-PCA reporter. The EGFP-C and EGFP-N fusion partners are labeled on the top and left of each image.

To study the dynamics of CtBP dimer formation in response to the metabolic status we initially sought to confirm CTBP heterodimer interaction in SKOV3 cells through different approaches. We constructed HA-CtBP1 and FLAG-CtBP2 and performed immunoprecipitation assays to demonstrate the presence of the CtBP dimer in live cells. While the ectopically expressed HA-CtBP1 and Flag-CtBP2 showed approximately equal levels of expression, applying more of the anti-Flag antibody increased the pull-down efficiency of HA-tagged CtBP1 in OC cells (**Fig. 1A**). This dose-dependent pulldown assay indicates that HA-CtBP1 and Flag-CtBP2 are part of the same complex. We also performed BioID (Proximity-dependent Biotin Identification 38) with either CtBP1 or CtBP2 fused with BirA*, the biotin ligase. Both CtBP1 and CtBP2 were identified to be biotinylated using western blotting (**Fig. 1B and Supplementary Fig. 1A**), suggesting that CtBP1 and CtBP2 form a complex with the bait protein CtBP1/2-BirA* in SKOV3 cells. In addition, in the wild-type SKOV3 cell lysate, CtBP1 or CtBP2 coprecipitated with each other, as revealed by the LC-MS analysis (**Supplementary Fig. 1B and Supplementary Fig. 1C**). However, these assays couldn't prove that HA-CtBP1 and Flag-CtBP2 form dimers directly as reported in previous crystallography studies 28, 39. In comparison, the PCA assay requires the spatiotemporal juxtaposition of the two fragments of the reporter protein and is more reliable to disclose direct protein-protein interactions 40 (**Fig. 1C**). Thus, we applied CtBP-Gluc-PCA to directly observe the formation of the CtBP dimer 41. CtBP2 in fusion with either the Gluc N-terminus or C-terminus was transfected into 293 cells. CtBP2-Gluc-N/CtBP2-Gluc-C co-transfected cells showed a significant increase in the Gluc luminance signal compared to the negative controls. In addition, the data indicate that the formation of the CtBP2 homodimer was weak because the signal from CtBP2 homodimer is 10-fold lower than the signal emitted by the Zip-Zip dimer (**Fig. 1D**). MTOB has been reported to interact with CtBP, and has the potential to interrupt CtBP dimerization and corepressor function 39. Thus, we also tested the response of CtBP-Gluc-PCA reporters to MTOB treatment. As shown in **Fig. 1E**, a dose-dependent reduction in the CtBP-Gluc-PCA signal was observed, revealing that CtBP dimerization was dynamically regulated by MTOB.

Tetramerization of CtBP was recently reported, and multiple mutants encompassing several key residues destroy the tetramerization and repressive function 30. Among these mutants, tryptophan 318 in CtBP1 was shown to be a critical residue for tetramer formation and the repressive function 29, 39. This residue is also located within the NAD^+^/NADH binding domain and is conserved in CtBP2 as tryptophan 324 (**Fig. 1F**). Next, HA-CtBP1 was co-transfected with either the Flag-CtBP2 or Flag-CtBP2-W324G mutant into 293 cells. Both anti-HA and anti-Flag antibodies pulled down Flag-CtBP2 or HA-CtBP1, respectively, in the HA-CtBP1/Flag-CtBP2 co-transfected group. But the pull-down efficiency substantially reduced when the co-transfected Flag-CtBP2 contains the W324G mutation (**Fig. 1G and Supplementary Fig. 1D**). We further employed the EGFP-PCA assay in which CtBP was fused with either the EGFP N-terminus or C-terminus to directly view the formation of CtBP dimers. The formation of both the homodimers of CtBP1 and CtBP2 and the heterodimer between CtBP1 and CtBP2 was confirmed according to the reconstitution of the EGFP signal (**Fig. 1H**). Importantly, the CtBP2 homodimer is exclusively located in the cell nucleus, while the CtBP1 homodimer and the CtBP1 and CtBP2 heterodimers appears in both the cytoplasm and nucleus. Consistently, the introduction of the W318G/W324G mutation into the CtBP-EGFP-PCA reporter also abolished EGFP signal reconstitution (**Fig. 1H and Supplementary Fig. 1E**). These data suggest that heterodimer formation between CtBP1 and CtBP2 is partially abolished by the W324G mutation. Since the EGFP-PCA experiment is based on direct protein interaction, the detection of CTBP1 in the CTBP2 W324G mutant pulldown experiment, although reduced compared to the CTBP2 wt, suggests that CTBP1 can still be partially co-precipitated through the interaction with other proteins.

### The CtBP2-W324G mutant shows a distinct binding profile than wild-type CtBP2

**Figure 2:**
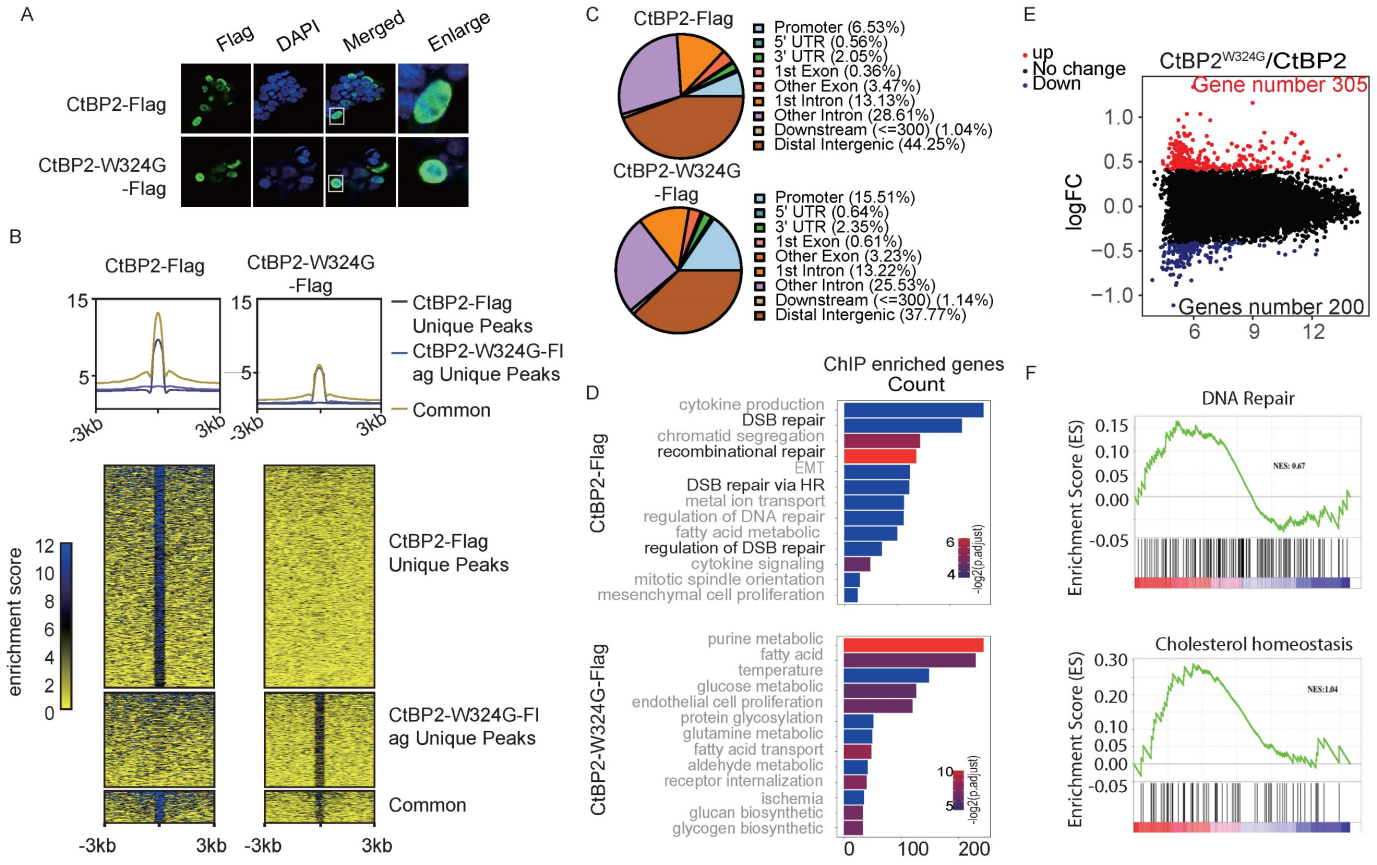
** Characterization of CtBP binding in OC cells.** A. Nuclear localization of CtBP2-Flag and CtBP2-W324G-Flag in SKOV3 cells transfected with the respective vectors. B. Global distribution of CtBP2-Flag and CtBP2-W324G-Flag in the SKOV3 genome, as characterized using ChIP-seq. The top panel shows the consensus signals from unique CtBP2-Flag peaks, unique CtBP2-W324G-Flag peaks and both within the flanking (+/- 3 kb) region of the peaks. The bottom panel shows the read distribution and displays clusters according to the uniqueness status. C. Distribution of the binding peaks of CtBP2-Flag and CtBP2-W324G-Flag at different genomic functional elements. D. Gene Ontology analysis of the genes whose promoters were bound to CtBP2-Flag or CtBP2-W324G-Flag. E. Volcano plot of differentially expressed genes between CtBP2-Flag and CtBP2-W324G-Flag. The number of upregulated and downregulated genes is also labeled. F. GSEA of DEGs between CtBP2-Flag- and CtBP2-W324G-Flag-transfected SKOV3 cells. DNA repair and cholesterol homeostasis are the two top-listed gene sets enriched in the CtBP2-Flag condition.

CtBP is a well-established corepressor and is critical for recruiting other corepressors, including many epigenetic modifiers. The currently available model indicates that the dimerization of CtBP is indispensable for its corepressor function. However, other studies also indicated that CtBP may regulate gene expression as a monomer. The genome-wide binding profiles of CtBP2 and the CtBP2-W324G mutant were measured to identify the genes that are specifically regulated by the CtBP dimer but not the monomer. Both CtBP2 and CtBP2-W324G were tagged with Flag and were transfected into SKOV3 cells. Induction of the expression of both vectors was confirmed using the anti-Flag antibody that was also used for chromatin immunoprecipitation (ChIP) in SKOV3 cells (**Supplementary Fig. 2A**). As expected, CtBP2 and the mutant protein were exclusively located in the nucleus (**Fig. 2A**). Globally, the binding strength of CtBP2-W324G was significantly reduced (**Supplementary Fig. 2B, Supplementary Table 1 and Supplementary Table 2**). Consistent with previous studies showing that CtBP functions as a corepressor by forming a dimer, more unique CtBP2 binding peaks were observed cross the whole genome than the CtBP2-W324G mutant (**Fig. 2B**). The binding strength of the mutant also decreased significantly compared to the wild-type CtBP (**Fig. 2B**). For the small fraction of peaks displaying both CtBP2 and mutant binding, the average CtBP2 binding strength was still higher than CtBP2-W324G binding strength (**Fig. 2B**). We further analyzed the consensus motifs enriched from the peaks bound to either CtBP2 or the mutant. Unexpectedly, CtBP2 and CtBP2-W324G mutant have many overlapped binding motifs and very few differentially enriched motifs were identified (**Supplementary Fig. C**). Since CtBP does not contain the known DNA binding domain and requires other mediators for its chromatin binding, these data suggest that the mutant still maintains its ability to interact with other mediators that recruit CtBP to chromatin. Another unexpected observation was that mutant CtBP2 exhibited greater binding to the gene promoter region (CtBP2 mutant 15.51% versus CtBP2 6.53%) and the first exon region (CtBP2 mutant 0.61% versus CtBP2 0.36%) than the wildtype CtBP2 (**Fig. 2C**). Consistently, the percentage of binding peaks identified from the CtBP2 mutant at the +/- 1 kb region of the TSS (transcription start site) was significantly higher than that of wild-type CtBP2 (+/‒1 kb, **Supplementary Fig. 2D)**. But the absolute number of genes bound by the CtBP2 mutant was significantly less than that bound by wild-type CtBP2 (7479 vs. 12456). A Gene Ontology analysis was performed to analyze the genes whose promoters contain binding peaks enriched for either CtBP2 or the CtBP2 mutant and to understand the effect of the loss of CtBP2 dimerization on the functions of the target genes. Consistent with our previous results, CtBP2 binds to genes enriched in DNA repair 19. Surprisingly, the CtBP2 mutant binds to genes enriched in functions related to cellular metabolic pathways (**Fig. 2D**). Next, both CtBP2 and the CtBP2 mutant were ectopically expressed in SKOV3 cells, and the change in gene expression was measured using RNA-seq to further understand the effect of the W342G mutation on CtBP2 function as a transcription factor. Consistent with our expectation, more genes were upregulated in the CtBP mutant-transfected cells (305 vs. 200) (**Fig. 2E**). These data suggest that the chromatin-binding defect in the CtBP2-W324G mutant correlates with its loss of function in repressing target gene expression. By analyzing the DEGs using GSEA, some high scoring gene sets were identified that matches the previously reported functions of CtBP, such as cholesterol homeostasis (**Fig. 2F**) 13, 19. DNA repair was also among the top hits of gene sets, as expected (**Fig. 2F**). In the CtBP mutant-enriched gene sets, the response to alpha-interferon and hypoxia scored the highest among many of the cellular metabolic functions (**Supplementary Fig. 2E**).

### The CtBP2 mutant loses the function of repressing DDR genes

**Figure 3:**
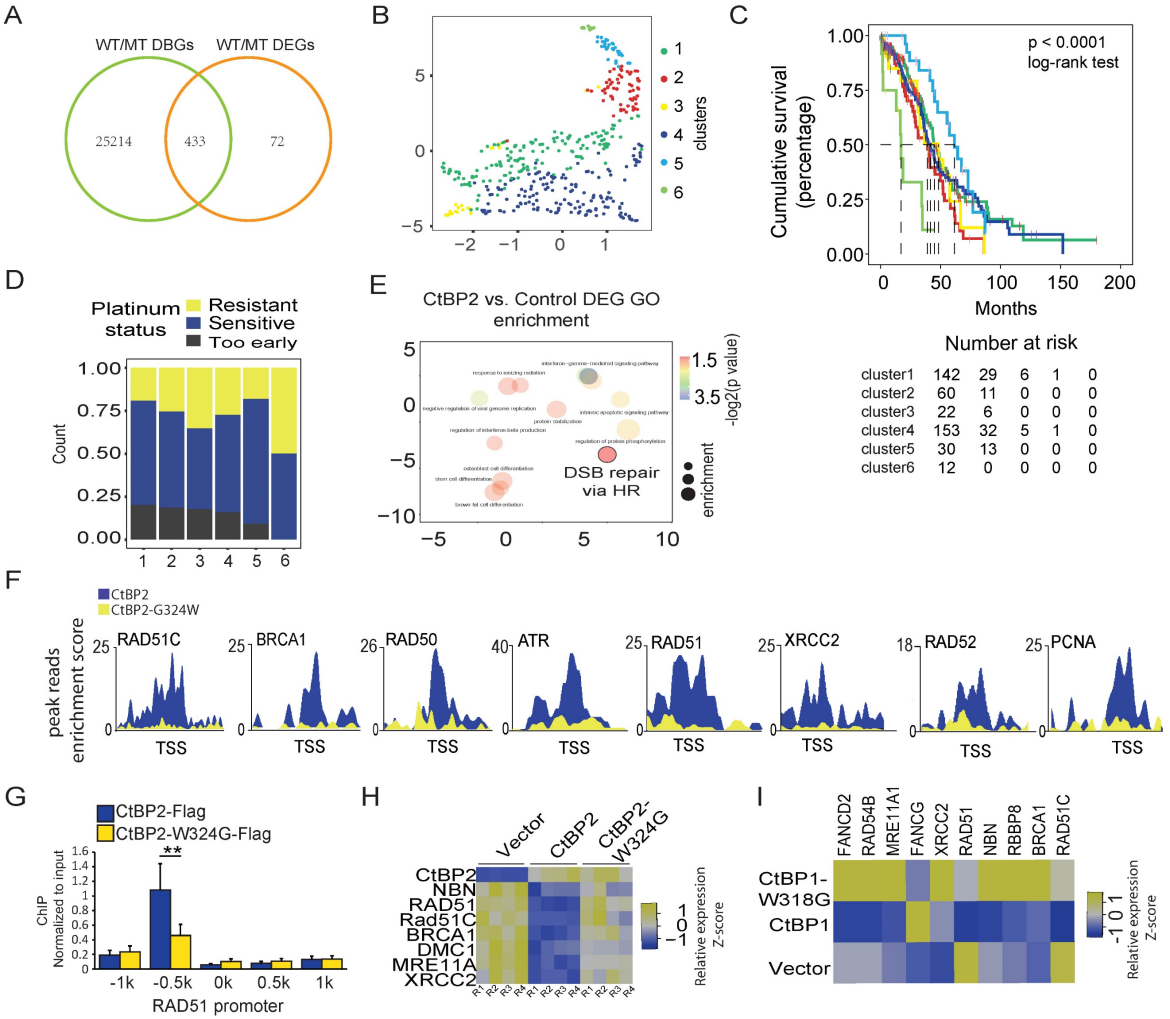
** CtBP regulates the expression of DDR genes in OC cells.** A. Venn diagram of differentially binding genes (DBGs) and differentially expressed genes (DEGs) between CtBP2- and CtBP2-W324G-transfected SKOV3 cells. B. Principal clustering analysis (PCA) of the groups of patients identified by a consensus clustering analysis of the overlapping genes between DBGs and DEGs in clinical samples (TCGA, OC cohort). C. The cumulative survival analysis of the groups of patients identified in B, the case number table is shown at the bottom. D. Analysis of the platinum sensitivity of the groups of patients identified in B. E. Gene Ontology analysis of the DEGs between the SKOV3 cells transfected with either the control vector or the CtBP2 overexpression vector. F. ChIP-seq read distribution at the TSS of the DDR gene loci. Blue indicates the binding peaks of CtBP2-Flag, and yellow indicates the binding peaks of CtBP2-W324G-Flag. G. Real-time PCR validation of CtBP2-Flag and CtBP2-W324G-Flag binding to the RAD51 gene promoter region (+/- 1 kb). H. Real-time PCR detection of DDR gene expression upon either wild-type CtBP2 or CtBP2-W324G overexpression. R1 to R4 represent four biological replicates. The yellow/blue heatmap scale indicates the log2 fold change. I. Real-time PCR detection of DDR gene expression upon either wild-type CtBP1 or CtBP1-W318G overexpression. The yellow/blue heatmap scale indicates the log2 fold change. Values are based on three biological replicates.

Considering the notable change in the gene repression function of the CtBP2-W324G mutant, we wondered whether this change is clinically relevant. Four hundred thirty-three genes that were identified as the most significant DEGs (p<0.001, FDR<0.05) and the genes with the most significant differential CtBP binding (p<0.001, FDR<0.05) between CtBP and CtBP-W324G were selected for further analysis (**Fig. 3A and Supplementary Table 5**). A consensus clustering analysis of the expression of these 443 genes in clinical samples (TCGA, ovarian cancer cohort) identified 6 groups of OC tumors (**Fig. 3B and Supplementary Fig. 3A**). Interestingly, these 6 groups of tumors showed significant differences in patient survival, with Group 6 having the worst prognosis and Group 1 having the best prognosis (p<0.0001) (**Fig. 3C**). Since DDR-related functions were the main enriched GO terms in the wild-type CtBP ChIP-seq analysis (**Fig. 2C**), we subsequently assessed whether these 6 groups of tumors showed any difference in platinum sensitivity. Again, a gradual increase in platinum resistance or decrease in platinum sensitivity was observed from Group 1 to Group 6 (**Fig. 3D**). This finding is consistent with the survival status of these 6 groups of patients. Platinum-based chemotherapy targets the DNA and the activity of the DDR pathways, especially the homologous-recombination dependent DNA repair pathway (HDR), which is negatively correlated with platinum sensitivity. The 433 potential wild-type CtBP target genes included many DDR genes (**Supplementary Table 5**) and the Gene Ontology analysis revealed that the DNA damage response is the main function associated with these genes as expected (**Supplementary Fig. 3B**). Moreover, DNA double-strand break (DSB) repair via HDR was one of the enriched GO terms in CtBP2-overexpressing SKOV3 cells (**Fig. 3E**). The CtBP2 mutant did not bind to the TSS of these HDR-related genes (**Fig. 3F and Supplementary Fig. 3C**). For instance, the representative HDR-related gene RAD51 was validated to lack CtBP2 mutant binding at the promoter (**Fig. 3G**). The expression of these HDR genes was also repressed by wild-type CtBP2 but not mutant CtBP2 (**Fig. 3H**). Surprisingly, wild-type CtBP1 overexpression in SKOV3 cells repressed HDR-related gene expression, as we predicted, but mutant CtBP1 instead upregulated the expression of many of these HDR-related genes (**Fig. 3I**). A possible explanation is that CtBP1 does not contain an NLS, and the overexpressed CtBP1 in the cytoplasm may restrict endogenous CtBP2 from entering the nucleus by forming a heterodimer.

### The CtBP status correlates with OC cell sensitivity to cisplatin

**Figure 4:**
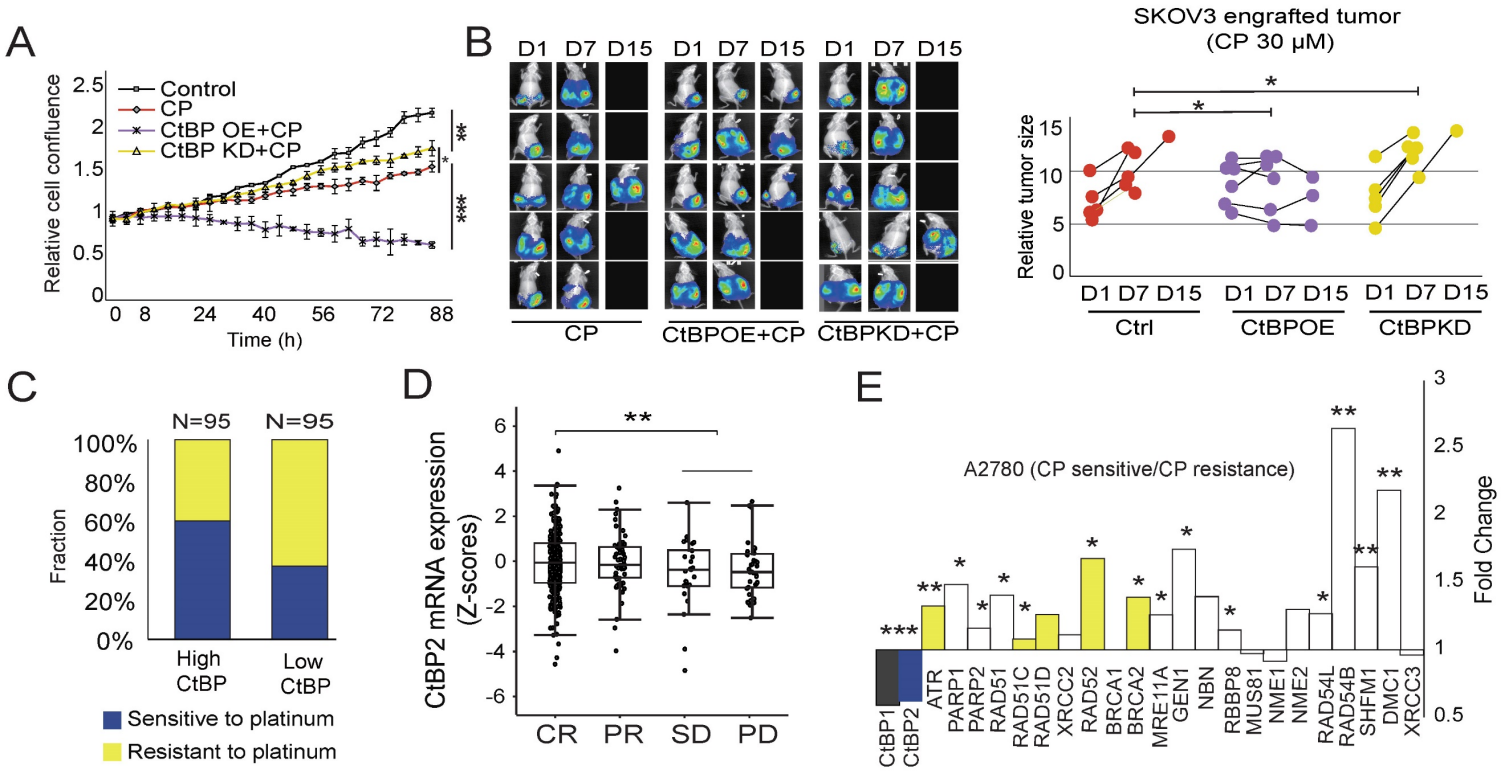
** Correlation between cisplatin (CP) sensitivity and the CtBP status in OC.** A. Measurement of the growth of SKOV3 cells subjected to cisplatin (CP) treatment. CtBP2 was either over-expressed (CtBP OE) or knockdown (CtBP KD), respectively. Cells without any treatment were used as the negative control. Error bars represent the SD from 3 biological replicates. * p<0.05, ** p<0.01, and *** p<0.001. B. Left panel, Luminance signal captured from tumor-engrafted mice. Images of each mouse were captured 3 times at D1, D7, and D15. The three groups of mice were engrafted with SKOV3 tumor cells transfected with the CtBP over-expression, CtBP knockdown, or empty vectors. Cisplatin (CP) was i.p. injected every other day at a dose of 5 mg/kg. Right panel. The plot of the luminance signal recorded from each mouse at different time points. ** p<0.01. C. Comparison of cisplatin (CP) sensitivity between high CtBP expression and low CtBP expression tumor samples (top and bottom 95 samples) extracted from OC dataset in the TCGA cohort. D. Comparison of CtBP2 mRNA expression in samples from patients with OC in the TCGA cohort. The patient was classified as CR, PR, SD, or PD based on their response to platinum drug treatment. E. Analysis of CtBP1, CtBP2, and DNA repair gene expression in cisplatin-sensitive and cisplatin-resistant A2780 cells. The original data is extracted from GSE33482. Among the DNA damage repair genes, the genes that are validated in CtBP2 ChIP assay (**Fig 3F and Supplementary Fig. 3C**) are in yellow.

Next, we either overexpressed CtBP2 or knocked down CtBP2 in SKOV3 cells and observed whether the change in CtBP expression alters the cell response to cisplatin. As expected, 30 µM cisplatin treatment significantly hindered SKOV3 cell growth. Overexpression of CtBP further significantly increased the sensitivity to cisplatin treatment, with a strong inhibition of cell growth (**Fig. 4A**). In contrast, CtBP knockdown had a marginal effect on cell proliferation in response to cisplatin treatment (**Fig. 4A**). Next, we generated a subcutaneous tumor-engrafted mouse model to validate the correlation between the CtBP status and cisplatin sensitivity. The tumors from SKOV3 cells with ectopic CtBP overexpression were more sensitive to cisplatin treatment, and the average tumor volume shrank after cisplatin treatment (**Fig. 4B**). By checking the expression of RAD51 and γH2AX, we further confirmed that CtBP OE profoundly influences the DNA damage repair activity in tumors under treatment by cisplatin (**Supplementary Fig. 4**). In comparison, the tumors from SKOV3 cells with CtBP knockdown showed similar or even faster tumor growth during cisplatin treatment compared to the control group without treatment (**Fig. 4B**). To further confirm that the CtBP status modulates tumor sensitivity to platinum treatment in clinical samples, the TCGA OC cohort tumor samples were divided to CtBP high (95 samples) and CtBP low (95 samples) expression groups according to the median expression level of CtBP. In the CtBP high expression group, approximately 60% of patients showed sensitive responses to cisplatin treatment compared to only 30% of patients in the CtBP low expression group (**Fig. 4C**). In addition, patients who achieved a CR (complete response) exhibited significantly higher CtBP2 mRNA expression than the SD and PD groups (**Fig. 4D**). In support of our conclusion that CtBP represses the drug resistance of ovarian cancer cells, the pair-wise comparison between cisplatin-resistant and cisplatin-sensitive A2780 cancer cells disclosed that cisplatin-resistant cells significantly downregulated both CtBP1 and CtBP2 (**Fig. 4E**). Importantly, nearly all DDR genes were upregulated in cisplatin resistant A2780 cells (**Fig. 4E**) and many of these genes have CtBP binding at their promoter (genes in yellow, **Fig. 3F and Supplementary Fig. 3C**).

### Cisplatin treatment depletes NADH and impairs the dimerization of CtBP *in vivo*

**Figure 5:**
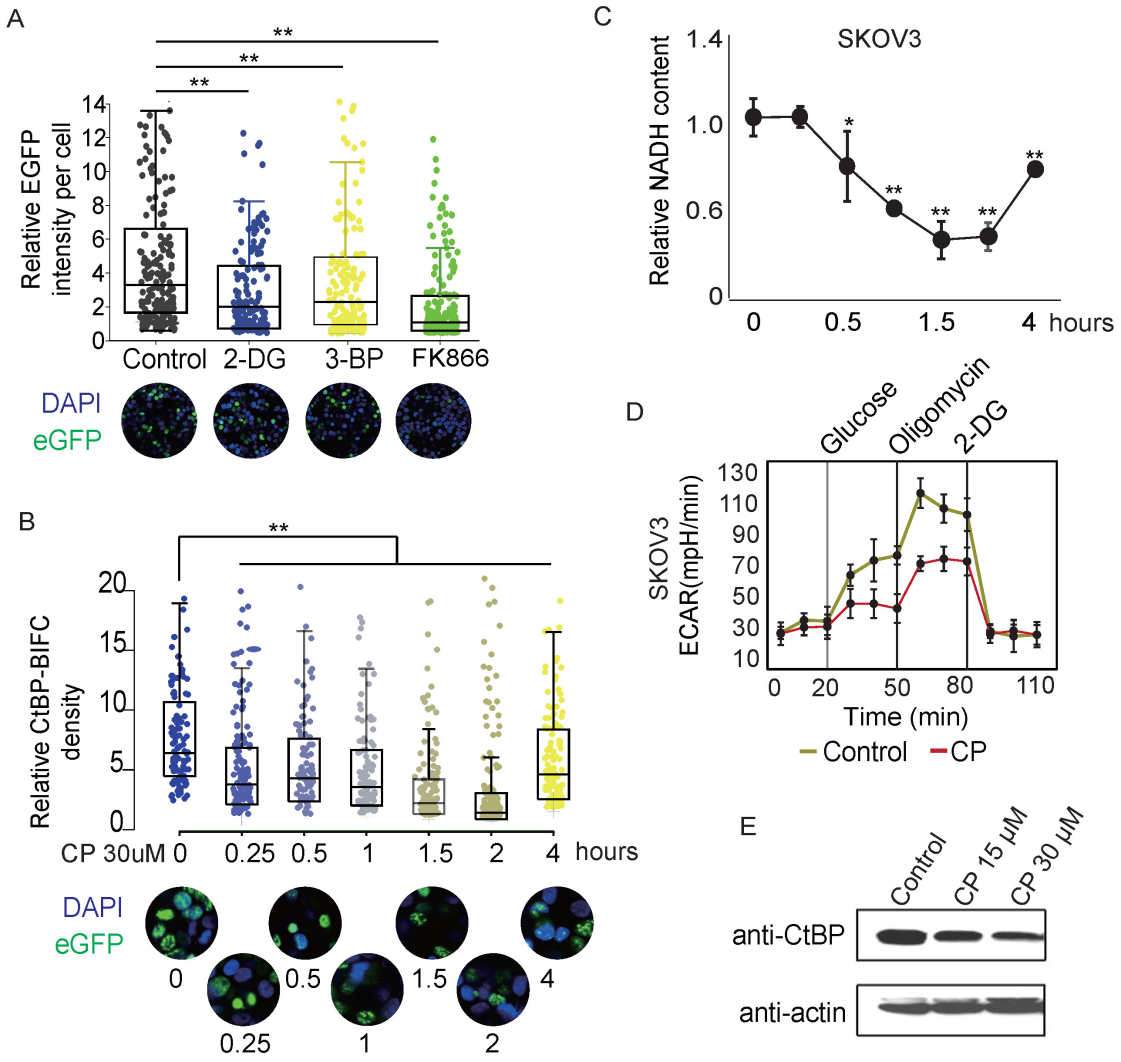
** CtBP dimerization is regulated by metabolic activities.** A. Dot-box plot of the EGFP-PCA of CtBP2 dimerization in response to the chemicals that interfere with metabolic activity. Each dot represents the EGFP signal from a single cell. The bottom panel shows representative images of each treatment condition. B. Dot-box plot of the EGFP-PCA of CtBP2 dimerization in cells treated with 30 µM cisplatin (CP) for different times. The bottom panel shows representative images for each treatment time point. C. NADH quantitation in SKOV3 cells subjected to cisplatin (CP, 30 µM) treatment for different time. Error bars represent the SD from 3 biological replicates. * p<0.05 and ** p<0.01. D. Quantitation of CtBP levels in cells treated with cisplatin (CP) for 24 hours using western blotting. E. ECAR measurements in SKOV3 cells treated with cisplatin (CP) (30 µM) for 2 hours. Error bars represent the SD from 3 biological replicates.

NADH and NAD are both known to mediate the formation of CtBP dimers 28, 42-44. Some studies have proposed that NADH has a higher affinity for binding to CtBP than NAD^+^. Therefore, CtBP might be a metabolic sensor. However, none of the previous studies indicated that NADH or NAD^+^ alters the dynamics of CtBP dimerization. Here, we used the CtBP-EGFP-PAC reporter to observe whether NADH or NAD^+^ influences the dimerization of CtBP in live cells. SKOV3 cells were treated with the glycolysis inhibitors 2-DG or 3-BP and the NAD^+^ synthesis pathway inhibitor FK866 to induce changes in NAD^+^ or NADH levels 43. All three chemicals significantly reduced the recombinant EGFP signal (**Fig. 5A**), suggesting alteration of NADH/NAD+ metabolism influences the CtBP dimer. Cisplatin treatment is a standard first-line chemotherapy for OC. Previous study has found a synergistic effect between cisplatin and PARP inhibitors in treating cancer 45, suggesting cisplatin treatment activates PAPR. Since PARP activation is associated with the depletion of NAD+ and NADH 46, we suspect if cisplatin inhibits CtBP dimerization through PARP activation. Consistent to our expectation, we observed that acute cisplatin treatment decreased the signal from the EGFP-PAC-CtBP dimerization reporter in a time-dependent manner (**Fig. 5B**). Measurement of intracellular free NADH levels indicated that the free NADH level gradually decreased following cisplatin treatment (**Fig. 5C**). The NADH/NAD^+^ ratio was also substantially decreased by cisplatin treatment in SKOV3 cells (**Supplementary Fig. 5A**), suggesting cisplatin influences the NADH metabolism. To study how cisplatin influences NADH metabolism, the cells were treated by cisplatin for 2 hours and the global alteration of the intracellular metabolism was assessed by Seahorse bioanalyzer. Interestingly, cisplatin-treated cancer cells showed a decreased ECAR (extracellular acidification rate), suggesting the loss of glycolytic activity. Instead, the cisplatin treated cells showed no obvious difference in OCR (oxygen consumption rate), suggesting brief exposure of cells to cisplatin didn't influence the respiration activity (**Fig. 5D** and **Supplementary Fig. 5B**) 47. These results indicate that cisplatin targets glycolysis pathway and leads to the brief decreasing of intracellular NADH, which has direct influence on CtBP dimerization. If the exposure of cells to cisplatin extended over 24 hours, CtBP is degraded via the ubiquitin-proteasome pathway as reported previously (**Fig. 5E**) 48. Together, these observations suggest cisplatin breaks down CtBP dimerization abruptly through influencing the glycolysis activity and long-term exposure of cells to cisplatin results in the degradation of CtBP.

### Metformin enhances the DNA damage effect of cisplatin

**Figure 6:**
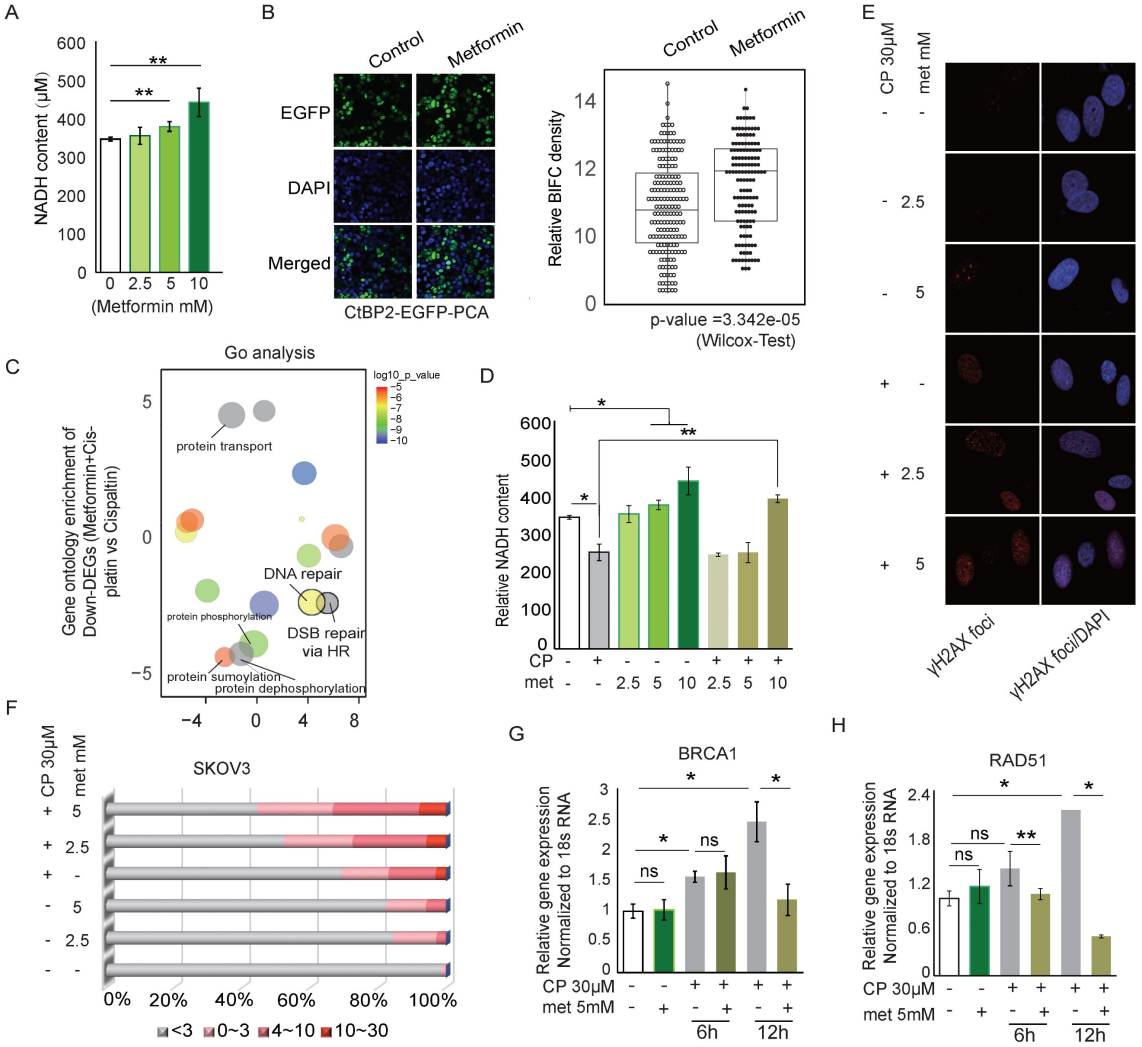
** Metformin modifies the CtBP dimerization status and regulates the DNA damage response.** A. Quantitation of NADH levels in SKOV3 cells treated with different concentrations of metformin (met). B. EGFP-PCA assay of CtBP2 dimerization in SKOV3 cells treated with metformin (met). Left panel, Representative images of EGFP signals. Right panel, Quantitation of the EGFP signal from 200 individual cells under each condition. The p value indicates a significant difference between the control and metformin (Met) treatment groups. C. Display of the GO terms enriched among the downregulated genes in SKOV3 cells treated with metformin and cisplatin compared to cells treated with cisplatin alone. D. Quantitation of NADH levels in SKOV3 cells treated with different doses of metformin (met), cisplatin or metformin (met) plus cisplatin (CP). Error bars represent the SD from 3 biological replicates. * p<0.05 and ** p<0.01. E. Image of DNA damage detected using anti-γH2AX staining in SKOV3 cells treated with different doses of metformin (met), cisplatin (CP), or metformin (met) plus cisplatin (CP). F. Quantitation of the number of anti-γH2AX foci in SKOV3 cells treated with different doses of metformin (met), cisplatin (CP), or metformin (met) plus cisplatin (CP), as shown in E. G. and H. Real-time PCR analysis of BRCA1 and RAD51 expression in SKOV3 cells treated with metformin (met), cisplatin (CP) or metformin (met) plus cisplatin for different time.

Previous studies have observed that metformin increases the sensitivity of lung cancer, triple-negative breast cancer, and brain cancer to platinum therapy by targeting the AMPK-mTOR pathway 49-51. Since metformin inhibits Complex I of the ETC (electronic transportation chain) in mitochondria and directly interrupts the oxidation of NADH, we hypothesized that metformin preserves CtBP dimerization and thus might enhance cisplatin-induced DNA damage by promoting CtBP dimerization-dependent repression of DDR genes. As expected, metformin increased intracellular NADH levels in SKOV3 cells in a dose-dependent manner (**Fig. 6A**). Consequently, metformin significantly increased the dimerization of CtBP (p=3.342e-05, Wilcox test), based on the signal from the CtBP2-EGFP-PAC reporter (**Fig. 6B**). Next, the differentially expressed genes between the SKOV3 cells treated with cisplatin (30 µM) alone or cisplatin (30 µM) plus metformin (5 mM) were analyzed for enriched GO terms (**Supplementary Table 3 and Supplementary Table 4**). DNA repair or DNA double-strand break (DSB) repair via HR was among the top enriched GO terms, suggesting that metformin mainly modulates the DDR pathways when added to cells along with cisplatin (**Fig. 6C**). We further compared the NADH levels in cells treated with metformin, cisplatin, or both compounds. Although cisplatin reduced and metformin increased intracellular NADH levels, treatment with cisplatin plus 10 mM metformin significantly increased NADH levels compared to cisplatin treatment alone (**Fig. 6D**). In addition, an increased number of DSBs was observed in cells treated with both cisplatin and metformin compared to cells treated with cisplatin alone (**Fig. 6E**). Further analysis of the γH2AX foci in individual cells treated by metformin and cisplatin revealed dose-dependent accumulation of DNA damage foci (**Fig. 6F**). BRCA1 and RAD51 are two key genes involved in the DDR response. Metformin alone did not alter the expression of these two genes in SKOV3 cells. However, metformin combined with cisplatin significantly reduced the expression of the BRCA1 and RAD51 mRNAs in 12 hours (**Fig. 6G and 6H**). Consistent with these findings, many of the genes directly participating in the DSB repair pathway, such as NBN, MRE11A, XRCC2, and CTIP, were significantly downregulated by metformin, either alone or in combination with cisplatin (**Supplementary Fig. 6A**).

### Application of metformin and cisplatin in a preclinical OC model proves the therapeutic effect of the dual treatment

**Figure 7:**
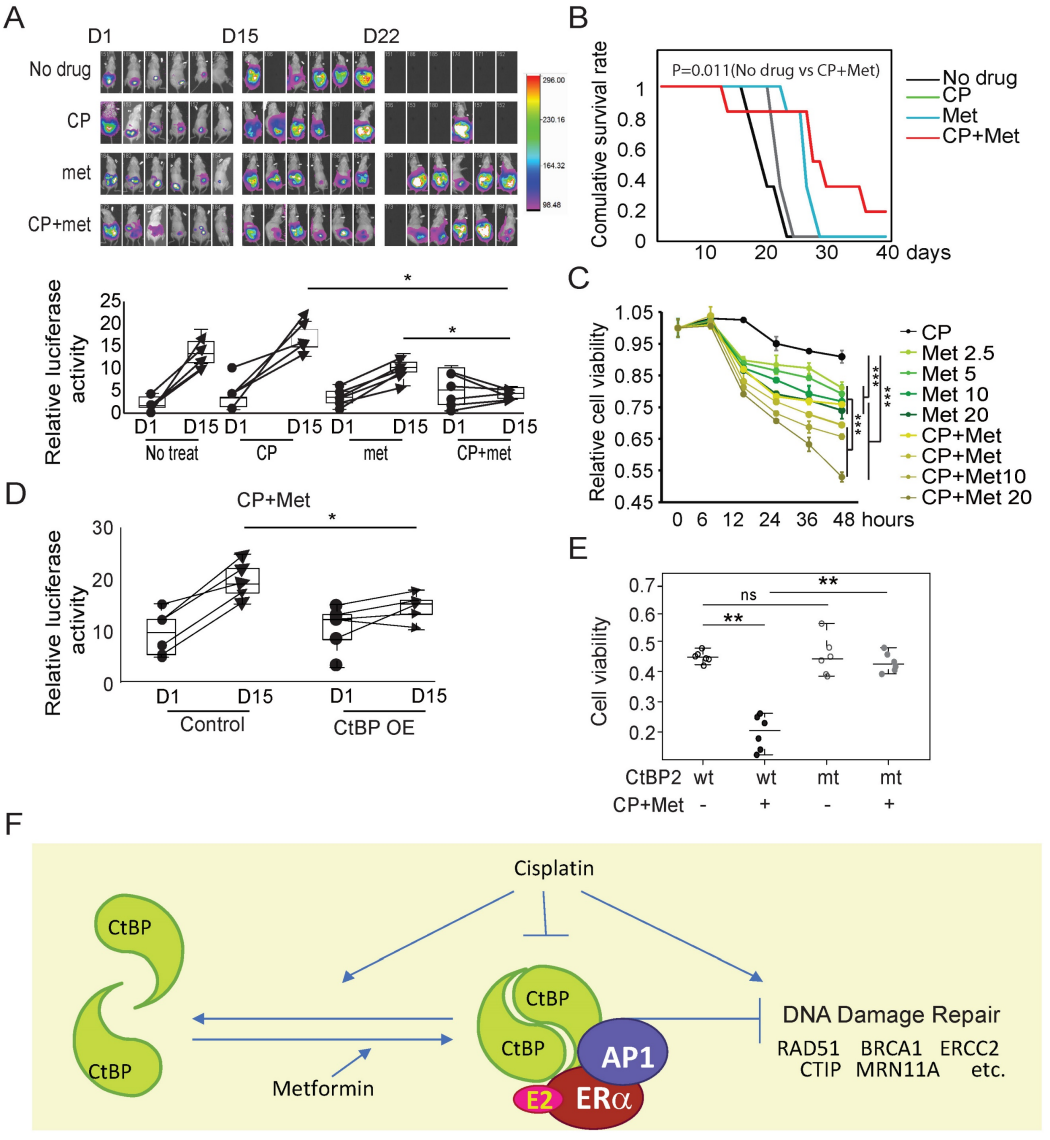
** Metformin enhances the therapeutic effect of cisplatin (CP) by promoting CtBP dimerization in OC.** A. Top panel, Luminance signal captured from tumor-engrafted mice. Images of each mouse were captured 3 times at D1, D15, and D22. The four groups of mice were engrafted with SKOV3 tumors and treated with cisplatin, metformin, or cisplatin plus metformin. Both drugs were i.p. (intraperitoneally) injected every other day at doses of 250 mg/kg metformin and 5 mg/kg cisplatin. Bottom panel, Plot of the luminance signal collected from each mouse at different time points. * p<0.05. B. Survival curves of the four groups of mice shown in A. p value was obtained using logrank test. C. SKOV3 cell viability after treatment with cisplatin (CP, 30 µM), metformin (met, 5 mM), or cisplatin (CP, 30 µM) plus metformin (met, 5 mM). *** p<0.001. D. Plot of the luminance signal collected from mice engrafted with tumors derived from SKOV3 cells with (CtBP-OE) or without (Control) CtBP over-expression. The images were captured at different time points, and images from D1 and D15 are shown to compare the change in the tumor luminance signal. * p<0.05. E. Dot plot of the viability of SKOV3 cells transfected with either CtBP2 or the CtBP2-W324G mutant and treated with cisplatin (30 µM) plus metformin (5 mM). Each dot represents one biological replicate. ns indicates no statistically significant difference. ** p<0.01. F. The model to illustrate the CtBP dimerization in response to metformin and cisplatin. Metformin promotes the dimerization of CtBP and cisplatin inhibits the dimerization and degrades CtBP. CtBP dimer represses the expression of DNA damage repair genes, but cisplatin induces the expression of these genes. In addition, our previous study indicated that ER and AP1 recruits CtBP to the DNA damage repair genes in OC cells.

A SKOV3 xenograft tumor model was established to further prove that metformin improved the therapeutic effect of cisplatin on OC. Although the initial tumor volume, as represented by the luciferase intensity, was similar across the no treatment, cisplatin-treated, metformin-treated, and cisplatin plus metformin-treated groups, metformin monotherapy showed a significantly better effect in limiting tumor growth than cisplatin, which produced a minimal effect. However, none of these monotherapies reached the therapeutic effect as shown in the cisplatin and metformin combination treatment group (**Fig. 7A, Group 4**). The survival of the tumor-harboring mice also proved that cisplatin in combination with metformin significantly extended the lifespan of the mice to a greater extent than the monotherapies with either cisplatin or metformin (**Fig. 7B**). The analysis of the viability of SKOV3 cells or ES-2 cells subjected to cisplatin plus metformin treatment showed much more significant cell death than cells treated with cisplatin alone or metformin alone (**Fig. 7C and Supplementary Fig. 7A**). The therapeutic effect of cisplatin plus metformin on SKOV3 xenograft tumors with the ectopic overexpression of CtBP2 was further evaluated to confirm that the ability of metformin to enhance the effect of cisplatin was partially mediated by CtBP. Compared to the tumors without CtBP2 overexpression, the CtBP-overexpressing tumors showed a significantly better response to cisplatin (30 µM) plus metformin (5 mM) dual treatment within a brief period (7 days) of treatment (**Fig. 7D**). Any single drug treatment showed no significant effect of curing in this SKOV3 engrafted tumor model. Moreover, only the cells with ectopic expression of wild-type CtBP2, but not the dimerization-defective CtBP2-W324G mutant, showed a significant reduction in cell viability upon treatment with cisplatin plus metformin (**Fig. 7E**). Comparable results were also replicated in cells with ectopic expression of either the CtBP1 or CtBP1-W318G mutant (**Supplementary Fig. 7B**). In conclusion, our data fit a model in which CtBP dimerization is enhanced by metformin, which antagonizes cisplatin resistance due to the degradation or inhibition of CtBP dimerization by cisplatin in OC. Together with our previous data 19, metformin-induced dimerized CtBP might form a complex with ligand-bound ER and be recruited to DDR genes as a repressor through AP1 (**Fig. 7F**).

## Discussion

Since the first observation of the CtBP dimer using crystallography twenty years ago by Kumar et al. 28, several studies have investigated the structural characteristics of the CtBP dimer. Recent studies have also proposed that the CtBP dimer further propagates into a tetramer as a functional unit. Disruption of the tetramer without influencing dimer formation modulates CtBP function *in vivo* 30. However, all of these previous studies of CtBP oligomers were based on *in vitro* analyses. Mutations that disrupt CtBP oligomerization *in vitro* have not been validated *in vivo*. Distinguishing the dimer from the tetramer *in vivo* is difficult, and no research tool is currently available. However, some approaches have been applied to distinguish the monomer from the dimer *in vivo*, such as coimmunoprecipitation, ligation-mediated PCR, and PCA. For the CtBP homodimer, the anti-CtBP antibody is not applicable in some of these methods unless CtBP is labeled by at least two types of tags. Even for the CtBP heterodimer, the anti-CtBP1 and anti-CtBP2 antibodies still have a certain level of cross-reaction, which prevents the application of these methods. We constructed CtBP expression vectors by fusing CtBP with either the HA/FLAG tags or the PCA reporter fragments to overcome these difficulties. By applying coimmunoprecipitation or PCA, we successfully determined the formation of a CtBP dimer *in vivo,* which was also subjected to regulation by the cell status. Notably, our observation might be affected by endogenous CtBP molecules. However, the negative effect of endogenous CtBP should have been minimized since our CtBP expression vectors contain a CMV promoter that promotes much higher expression of exogenous CtBP than endogenous CtBP.

Several studies have indicated that the NAD^+^ level, NADH level, or NADH/NAD^+^ ratio in the cytosol exerts a substantial effect on CtBP activity as a corepressor 18, 28, 52. The reason is that CtBP contains a well-conserved D2 hydroxy acid dehydrogenase (D2-HDH) domain, and NAD^+^/NADH are substrates fitting into the dehydrogenase pocket of CtBP. *In vitro*, purified CtBP shows dehydrogenase activity by catalyzing the conversion of NADH to NAD^+^ 53. However, while one study indicated that NAD^+^ and NADH binding to CtBP show no differences in promoting the CtBP interaction with its partner proteins such as E1A, other studies suggested that NADH binds with a much higher affinity to E1A, as well as the other CtBP interacting corepressors 28, 31, 35, 42. Functionally, many studies observed that increased NADH levels, but not NAD^+^ levels, enhances the repressor function of CtBP. The PCA approach allows us to directly address this question in live cells. In response to the perturbation of glycolysis activity, or the cisplatin treatment, we have shown the altered intracellular NADH by using a fluorescence probe. Our study supports NADH as the mediator of CtBP dimerization and by targeting NADH metabolism, the CtBP transcriptional repressor function could be blocked.

Platinum drug resistance in OC is the main reason for cancer relapse and metastasis. In this study, we documented a novel mechanism of drug resistance due to the loss of repression of DDR genes by CtBP. CtBP dimerization is a target of cisplatin treatment because of the depletion of NADH. We further demonstrated that the depletion of NADH is caused in partial by the reduced glycolysis activity. Interestingly, the mitochondria activity seems intact upon brief exposure of cells to cisplatin. Thus, we proposed that metformin, as a clinic drug, could serve as a NADH booster. Metformin is an ideal candidate due to its direct involvement in increasing intracellular NADH levels by blocking the TCA cycle coupled NADH consumption. Furthermore, metformin was found to increase drug sensitivity in several types of cancer, including OC 54. An even more interesting finding is that metformin alone prohibited OC cell proliferation without inducing cell death, similar to our observation, suggesting that the intact ETC and OXOPHOS are indispensable for OC cells 55. The combination of metformin and cisplatin exerted the greatest therapeutic efficacy in the SKOV3 xenograft mouse model. Compared to metformin alone, which slows tumor growth even better than cisplatin, the combination treatment obviously shrank the tumor over time, suggesting that metformin reversed cisplatin resistance in OC.

Our previous findings indicated that CtBP forms a complex with ER (estrogen receptor) to repress DDR genes. Thus, the postsurgical administration of hormone therapy to patients with OC might provide some benefit during follow-up chemotherapy. Estrogen has been shown to increase intracellular NADH levels in different tissues due to its ability to increase glycolysis activity 56-59. This finding has been confirmed in ER-positive breast cancer, which has a higher NADH level than ER-negative breast cancer, although both cancers are derived from the same tissue 60. Thus, CtBP might contribute to tumorigenesis in estrogen-responsive tissues such as the breast and ovary due to increased NADH abundance, since CtBP is a well-known repressor of tumor suppressor genes such as BRCA1, E-cadherin, and CDKN1A/2A etc. However, from a therapeutic perspective, drugs may disrupt CtBP dimerization by depleting NADH. Thus, supplementation with estrogen in patients with OC undergoing chemotherapy may not only increase the recruitment of CtBP/ER to DDR genes, as revealed in our previous report, but also increase CtBP dimerization, which is critical for the repression of target genes. Similarly, metformin could serve as an extra booster to further improve the dimerization of CtBP which leads to the repression of the DNA damage response and increases the chemotherapy response.

## Conclusions

In conclusion, our study presented a candidate biomarker, i.e., CtBP dimerization to indicate the sensitivity of OC patient to platinum drugs. In addition, CtBP dimerization could also serve as a therapeutic target to improve the sensitivity of OC patient to platinum drugs via applying the metabolic intervention drugs such as metformin.

## Supplementary Material

Supplementary figures.

Supplementary table 1: MT CHIP peaks.

Supplementary table 2: WT CHIP2 peaks.

Supplementary table 3: Gene expression WT MT.

Supplementary table 4: Gene expression CP CP+Met.

Supplementary table 5.

## Abbreviations

OC: Ovarian cancer
DDR: DNA damage repair
EOC: Epithelial ovarian cancer
CP: Cis-diamminedichloroplatinum
HR: Homologous-recombination mediated repair
FA: Fanconi anemia
CtBP: C-terminal binding protein
PCA: Protein fragment complementation assay
ChIP: Chromatin immunoprecipitation
ECAR: Extracellular acidification rate
OCR: Oxygen consumption rate
