# Supplementary material for: Metabolic modulation of CtBP dimeric status impacts the repression of DNA damage repair genes and the platinum sensitivity of ovarian cancer
Source: Int J Biol Sci. 2023 Apr 9;19(7):2081–96. doi: 10.7150/ijbs.80952 (PMC10158025; doi:10.7150/ijbs.80952)
Supplement: Supplementary file 1 — Supplementary figures. [file ijbsv19p2081s1.pdf]

Metabolic modulation of CtBP dimeric status impacts the repression of DNA damage repair genes and the platinum sensitivity of ovarian cancer

Jingjing Li[1,2,6,8], Yuan Wang[1,2,7,8], Li Wang[1,2,3,4],Dapeng Hao[1,2], Peipei Li[1,2], Minxia Su[4,5], Zhiqiang Zhao[1,2], Tianyu Liu[1,2,3,4], Lixin Tai[1,2,3,4], jinjian Lu[4,5], , Li-jun Di[1,2,3,4]

1.Department of biological sciences, Faculty of health sciences, University of Macau, Macau, PRC China
2.Cancer Center, Faculty of health sciences, University of Macau, Macau, PRC China
3.Institute of translational medicine, Faculty of health sciences, University of Macau, Macau, PRC China
4. Ministry of Education Frontiers Science Center for Precision Oncology, University of Macau, PRC China
5. Institute of Chinese Medical Sciences, University of Macau, Macau, PRC china
6. Current address: Jinming Yu Academician Workstation of Oncology, Affiliated Hospital of Weifang Medical University, Weifang, Shandong, China
7. Current address: State Key Laboratory of Respiratory Disease & National Clinical Research Center for Respiratory Disease, Guangzhou Institute of Respiratory Health, the First Affiliated Hospital of Guangzhou Medical University, Guangzhou Medical University, Guangzhou, China.
8. equal contribution

Corresponding to: Dr. Li-jun Di lijundi@um.edu.mo

Faculty of Health Sciences, University of Macau, Macau, SAR of People's Republic of China

E12-4009, Avenida da Universidade, Taipa,

Macau, China

Tel. 853-88224497

Fax. 853-88222314

Supplementary Figures and Supplementary Tables

Supplementary Fig 1

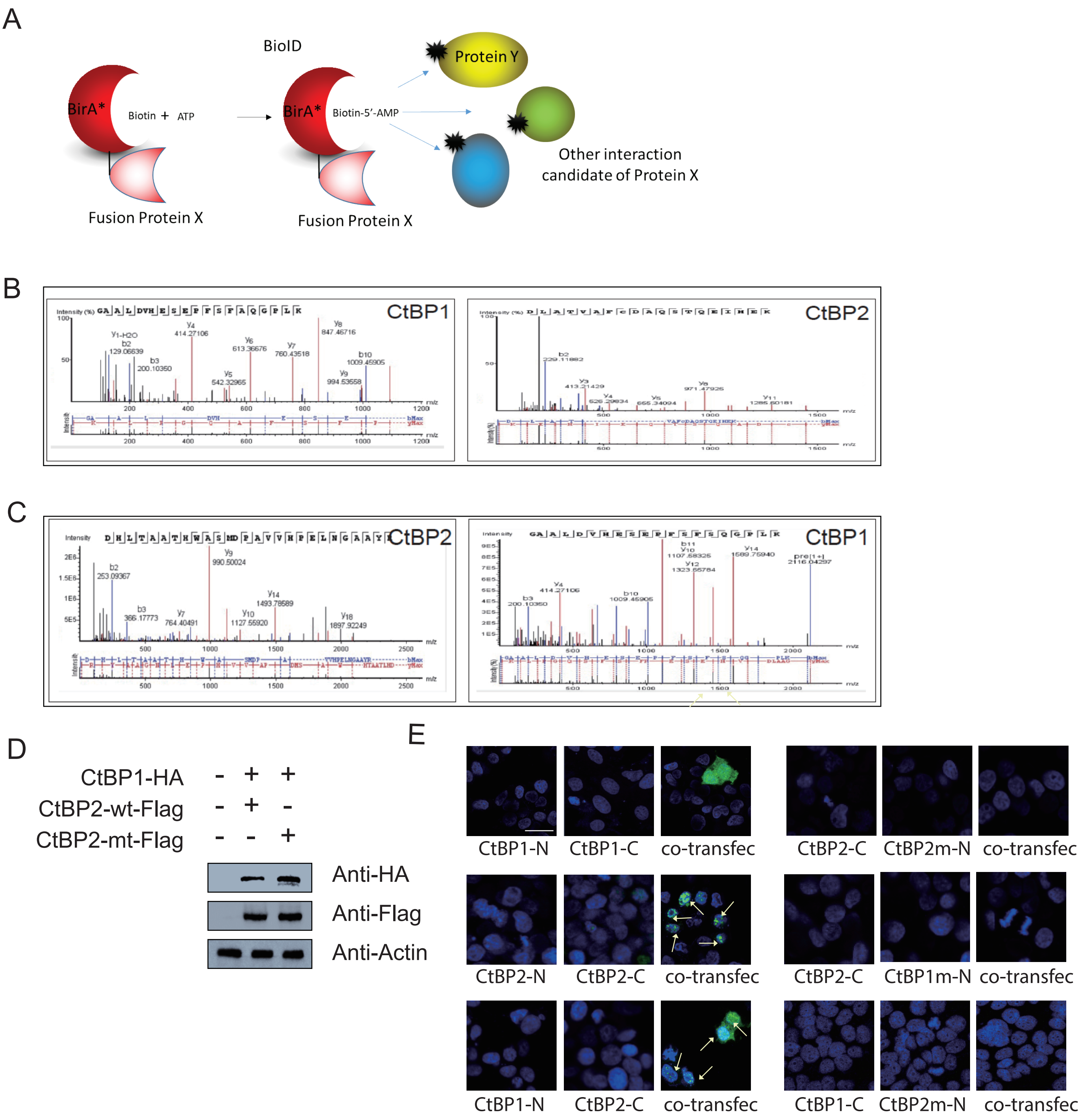

Supp. Fig1
A. Diagram showing the working mechanism of BioID technology. In this study, BioID is applied to validate the candidate interactions. B and C. Identification of CtBP1 and CtBP2 in the anti-CtBP1 and anti-CtBP2 pull-down lysates in SKOV3 cells using LC-MS. The representative peptide belongs to CtBP are shown according to the LC-MS signal. D. Western blot of the CtBP1-HA, CtBP2-Flag, CtBP2-W324G-Flag in SKOV3 cells for Co-IP assay. Actin serves as endogenous control. E. CtBP-EGFP-PCA assay with different combinations as indicated. For easy labeling, N and C represents the EGFP-N half and EGFP-C half of the PCA reporter. CtBP2m represent the CtBP2-W324G mutant and CtBP1m represent CtBP1-W318G mutant. DAPI was applied to stain the nucleus.

Supplementary Fig 2

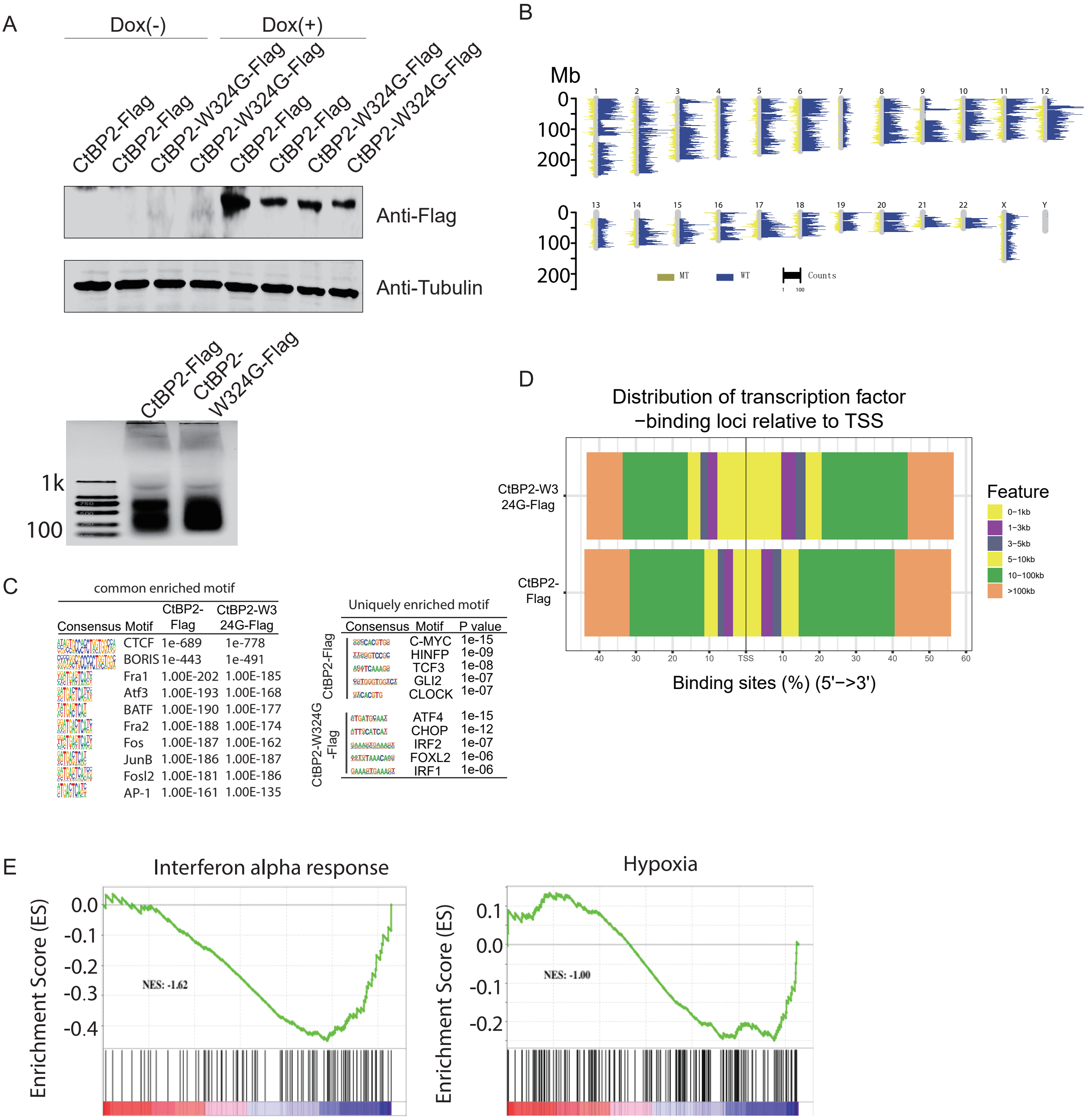

Supp.Fig2

A. Quality control of the cells used for ChIP-seq assay. CtBP2-Flag and CtBP2-W324G-Flag were induced by Dox (2ug/ml) for expression in SKOV3 cells. The sonication of formaldehyde (1%) fixed chromatin is also shown. B. Genome wide display of binding peaks from CtBP2-Flag (Dard Blue) and CtBP2-W324G-Flag (Yellow). C. The high score common (left) and unique (right) consensus motifs enriched from CtBP2-Flag and CtBP2-W324G-Flag peaks respectively. D. Distribution percentage of peaks from CtBP2-Flag and CtBP2-W324G-Flag samples at the promoter region.E. GSEA of DEGs between CtBP2-Flag and CtBP2-W324G-Flag transfected SKOV3 cells. Interferon Alpha response and Hypoxia are two top listed gene sets enriched in CtBP2-W324G-Flag condition.

Supplementary Fig3

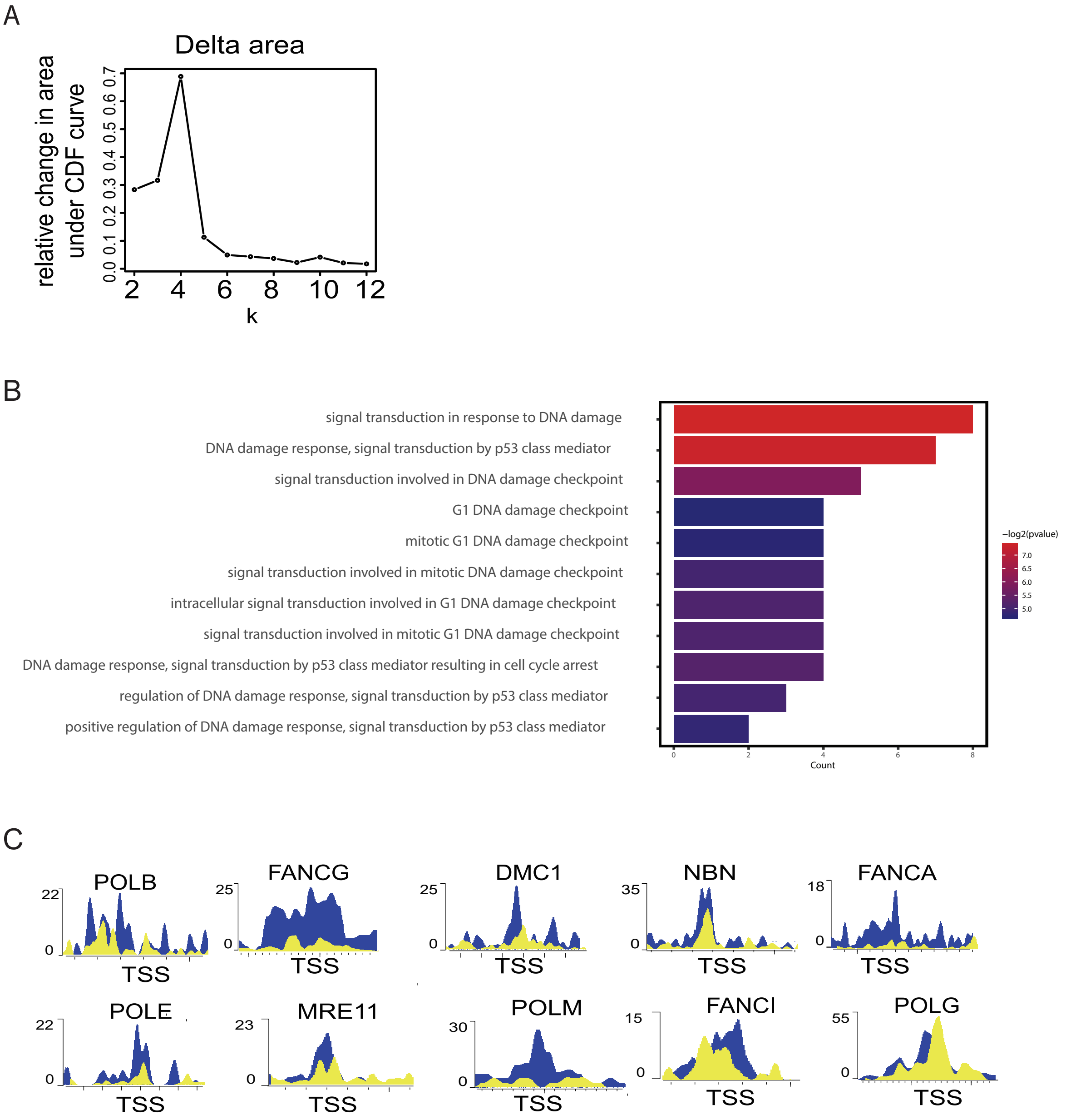

Supp.Fig3

A. Consensus curve : x-axis shows the number of sample groups and the y-axis represents the changes of CDF (Empirical cumulative distribution function). K=6 reached a steady state, that is the increasing number of groups does not change CDF much.B. Go analysis of the 433 genes enriched from DEGs with differential binding of CtBP2. C. The DNA damage repair genes with differential binding of CtBP2-Flag (Blue) and CtBP2-W324G-Flag (Yellow).

Supplement Figure 4

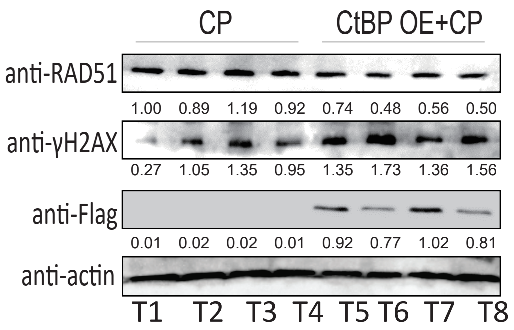

Supp. Fig4

Detection of RAD51 and γH2AX expression in response to cisplatin treatment in engrafted tumors formed by CtBP2 overexpression or empty vector. anti-Flag is to detect the tumor expresison of CtBP2. actin serves as endougenous control. T1-T4 are control tumors and T5-T8 are Tumors with CtBP OE.

Supplementary Fig 5

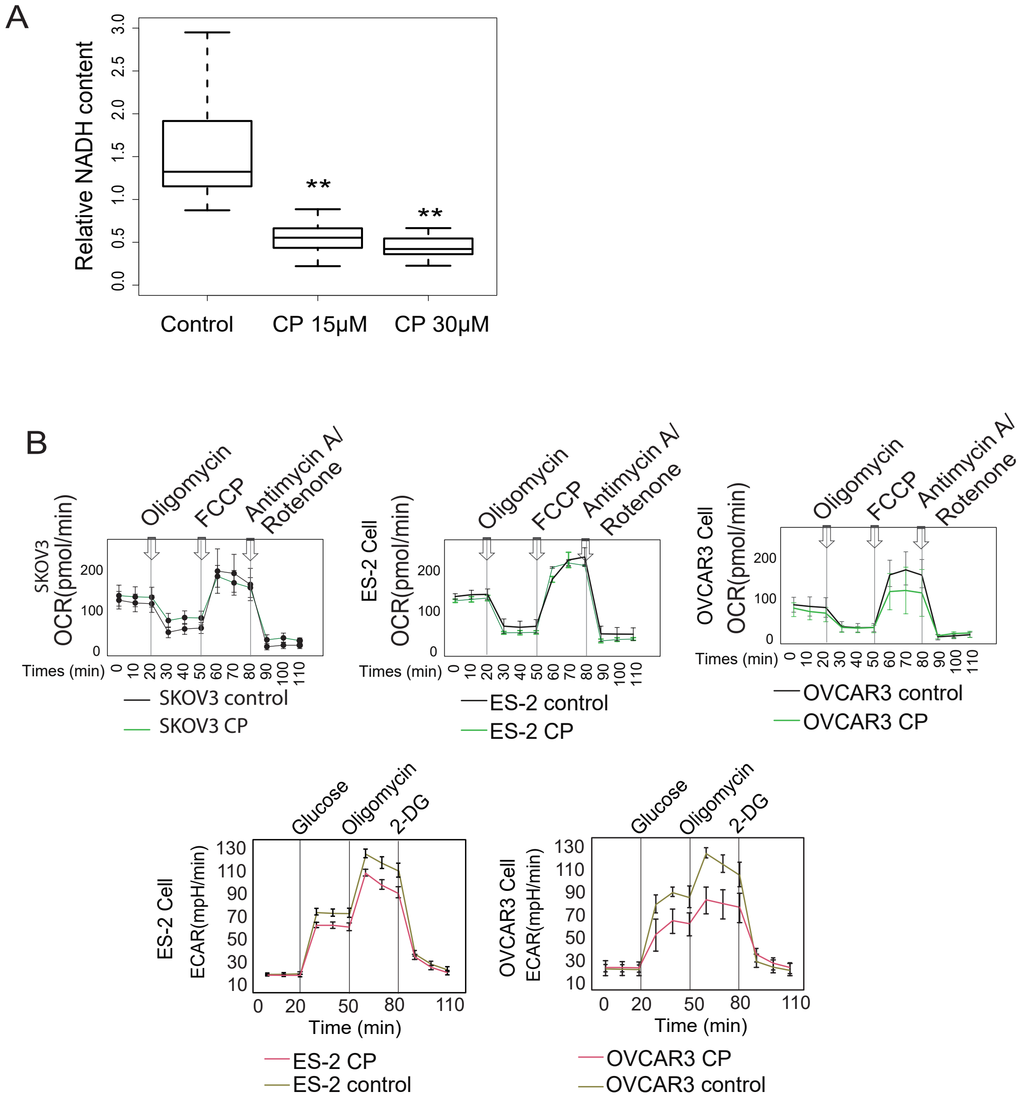

Supp.Fig5

A. Quantitation of NADH abundance in SKOV3 cells upon the cells are treated by Cisplatin with the dosage of 15 μM and 30 μM for 4 hrs. B. OCR (Oxygen Consumption Rate) measurement of SKOV3, ES-2 cell and OVCAR3 cell upon the Cisplatin (CP) treatment at dosage of 30 μM using the Seahorse bioanalyzer. Bottom are the ECAR measurement of ES-2 and OVCAR3 cells upon treatment by CP.

Supplementary Fig 6

A

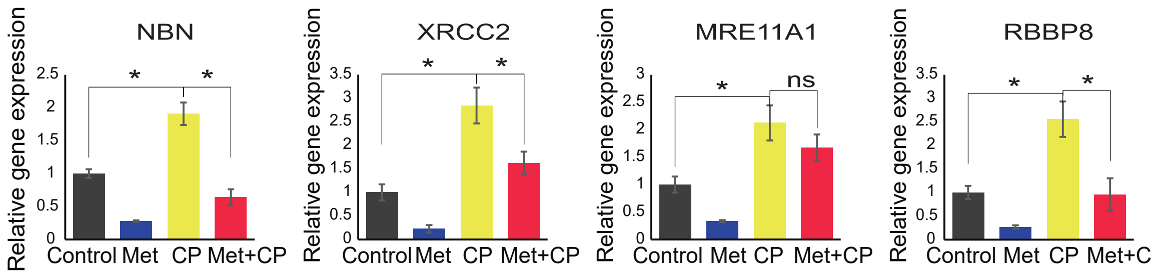

Supp. Fig6

A. Real-time PCR measurement of expression of the respective genes upon treatment by control, metformin (Met 5mM), cisplatin (CP, 30 µM), and Met (5mM) plus Cisplatin (CP, 30µ M).

Supplementary Fig 7

A

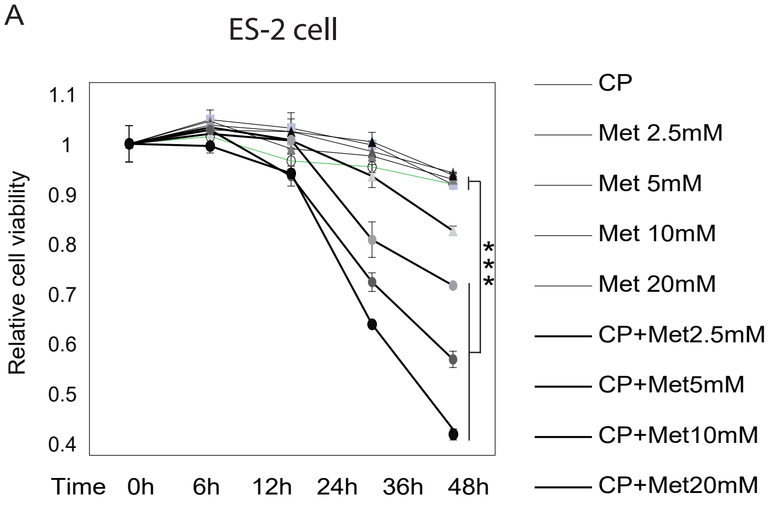

B

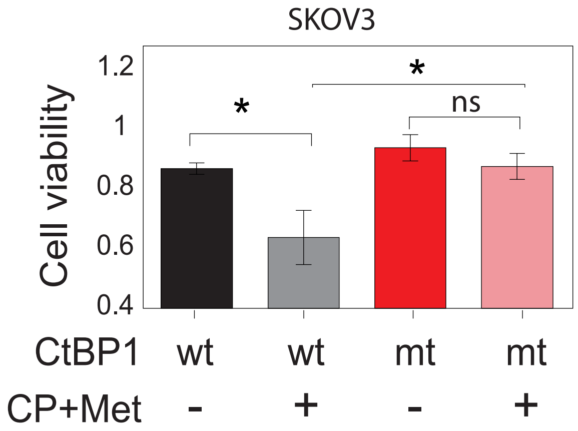

Supp. Fig7
A. ES-2 Cell viability measurement upon treatment by Cisplatin 30 μm, Metformin 5mM or Cisplatin plus Metformin combination. B. SKOV3 cells are transfected with either CtBP1(wt) or CtBP1-W318G mutant (mt) and the cells are also subjected to the treatment by cisplatin plus Metformin (CP, 30 μM plus Met, 5mM), the cell viability was measured and indicated along the y axis.
